# The pattern of xylan acetylation suggests xylan may interact with cellulose microfibrils as a twofold helical screw in the secondary plant cell wall of *Arabidopsis thaliana*

**DOI:** 10.1111/tpj.12575

**Published:** 2014-06-06

**Authors:** Marta Busse-Wicher, Thiago C F Gomes, Theodora Tryfona, Nino Nikolovski, Katherine Stott, Nicholas J Grantham, David N Bolam, Munir S Skaf, Paul Dupree

**Affiliations:** 1 Department of Biochemistry, University Of CambridgeTennis Court Road, Cambridge, CB2 1QW, UK; 2 Institute of Chemistry, University of Campinas-UNICAMPPO Box 6154, Campinas, SP, 13084-862, Brazil; 3 Institute for Cell and Molecular Biosciences, The Medical School, Newcastle UniversityNewcastle upon Tyne, NE2 4HH, UK

**Keywords:** xylan, acetylation, plant cell wall molecular architecture, cellulose interaction, *Arabidopsis thaliana*

## Abstract

The interaction between xylan and cellulose microfibrils is important for secondary cell wall properties in vascular plants; however, the molecular arrangement of xylan in the cell wall and the nature of the molecular bonding between the polysaccharides are unknown. In dicots, the xylan backbone of β-(1,4)-linked xylosyl residues is decorated by occasional glucuronic acid, and approximately one-half of the xylosyl residues are O-acetylated at C-2 or C-3. We recently proposed that the even, periodic spacing of GlcA residues in the major domain of dicot xylan might allow the xylan backbone to fold as a twofold helical screw to facilitate alignment along, and stable interaction with, cellulose fibrils; however, such an interaction might be adversely impacted by random acetylation of the xylan backbone. Here, we investigated the arrangement of acetyl residues in Arabidopsis xylan using mass spectrometry and NMR. Alternate xylosyl residues along the backbone are acetylated. Using molecular dynamics simulation, we found that a twofold helical screw conformation of xylan is stable in interactions with both hydrophilic and hydrophobic cellulose faces. Tight docking of xylan on the hydrophilic faces is feasible only for xylan decorated on alternate residues and folded as a twofold helical screw. The findings suggest an explanation for the importance of acetylation for xylan–cellulose interactions, and also have implications for our understanding of cell wall molecular architecture and properties, and biological degradation by pathogens and fungi. They will also impact strategies to improve lignocellulose processing for biorefining and bioenergy.

## Introduction

Xylan, a hemicellulose of plant secondary cell walls, and cellulose are the most abundant polysaccharides in plants. But, despite their importance, we do not understand how these two polymers are arranged in the cell wall and how they interact with each other. Hemicelluloses are thought to hydrogen-bond with cellulose, but the mechanism of interaction is not known. Recent progress has been made in understanding the primary cell wall structure (Park and Cosgrove, 2012), but we still lack molecular-scale models of the structure of lignocellulose of secondary cell walls (Cosgrove and Jarvis, 2012). The bonding between xylan and cellulose fibrils is likely to influence the strength and elasticity of walls, and contribute to the resistance of the walls to enzymatic degradation. Separation of xylan from cellulose is essential in many industrial processes. Knowledge of the molecular architecture of secondary cell walls will therefore be invaluable for the food, construction, paper and bioenergy sectors.

The functions and pattern of decorations on the xylan backbone are still not fully clear. The xylan backbone, composed of β-(1,4)-linked xylose units, carries various substitutions including acetylation, (4-*O*-methyl) glucuronic acid (GlcA), arabinose and others. These substitutions vary depending on the species and cell wall type (Ebringerová and Heinze, 2000; Scheller and Ulvskov, 2010; Koutaniemi *et al*., 2012). One of the functions of the decorations is likely to prevent digestion by microbial enzymes (Biely *et al*., 1986). The decorations are also likely to alter the interactions of the xylan with itself (thus maintaining solubility) and with other molecules in the wall, particularly cellulose and lignin. In grasses, arabinose residues carry the ferulic acid that allows cross-linking between xylan chains (Ishii, 1991) and linkages to lignin (Grabber *et al*., 2004). We recently found that in Arabidopsis, much of the xylan carries evenly spaced GlcA residues, and we named xylan with this pattern the major domain. We proposed that the decoration patterns may be important in allowing the xylan to interact with cellulose fibrils (Bromley *et al*., 2013). A minor domain of the same xylan molecules carries randomly spaced GlcA decorations, and will therefore have different properties for interaction with cell wall components (Bromley *et al*., 2013).

The GUX enzymes add GlcA to the xylan backbone (Mortimer *et al*., 2010; Rennie *et al*., 2012). GUX1 decorates the major domain of xylan, whereas GUX2 decorates solely the minor domain (Bromley *et al*., 2013). The *gux1 gux2* double mutant lacks any GlcA on xylan in the secondary cell walls (Mortimer *et al*., 2010; Bromley *et al*., 2013). Surprisingly, the mutants do not show xylem collapse, and the xylan appears to be functional. The presence of acetate groups maintains solubility and prevents the xylan from precipitating (Mortimer *et al*., 2010). The viability of *gux1 gux2* xylan indicates that acetylation is able to provide much of the function of the substitutions on xylan in secondary cell walls.

Acetylation changes the properties of polymers, such as interchain interactions and solubility (Pawar *et al*., 2013). Xylan acetylation may be important for secondary wall formation, and at least two families of proteins are involved in the addition of this decoration (Gille and Pauly, 2012). The RWA family of proteins (Manabe *et al*., 2011, 2013) are putative transporters of acetyl-CoA, providing a substrate for acetylation of sugars in the lumen of the Golgi apparatus. Less is known about how the substrate is transferred onto respective acceptors. It has been proposed recently that Eskimo1/TBL29, a TBL protein family member, is a putative xylan acetyl transferase (Xiong *et al*., 2013). Mutants in RWAs and in TBL29 lead to dwarfing, which is likely to result, at least in part, in the collapse of secondary cell wall xylem vessels because of reduced strength (Lefebvre *et al*., 2011; Manabe *et al*., 2013). Thus acetylation is important for xylan function.

The degree of xylan acetylation in dicots is estimated to be around 50% of xylosyl residues (van Hazendonk *et al*., 1996; Teleman *et al*., 2000, 2002; Evtuguin *et al*., 2003; Goncalves *et al*., 2008; Prozil *et al*., 2012; Xiong *et al*., 2013), but the distribution of acetyl groups along the chain has not been determined. It has previously been suggested that it is not random (Reicher *et al*., 1989). Here, we show that acetyl groups are preferentially present at every second xylosyl residue along the Arabidopsis xylan chain. Molecular dynamics simulations were used to investigate the ability of the decorated xylan to interact with the cellulose microfibril surfaces.

## Results

### Docking of acetylated xylan onto cellulose fibrils

**Figure 1:**
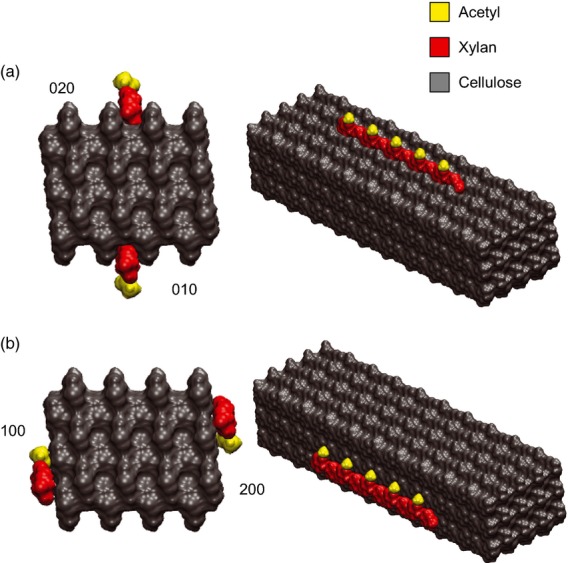
Model of acetylxylan interactions with a 24-chain cellulose microfibril. The end view and side view are shown. DP10 xylan chains with evenly spaced 2-*O*-Ac decorations at every two xylosyl residues were modelled as a twofold helical screw (2_1_). (a) Xylan chains placed on hydrophilic (010) and (020) faces. (b) Xylan chains placed on hydrophobic (100) and (200) faces.

To investigate *in silico* how acetyl esters could affect xylan–cellulose interactions, we docked models of acetylated xylan oligosaccharides, either with an even- or odd-spaced substitution pattern, to cellulose microfibrils. We found that acetyl esters on both sides of the ribbon (odd pattern) would hinder xylan–cellulose interactions on the hydrophilic (010) and (020) surfaces. Steric hindrance could be avoided if these decorations are spaced on alternate xylosyl residues to align on one side of the xylan ribbon, as shown in the model in Figure 1(a). If the xylan is layered on the hydrophobic (100) or (200) faces, then the xylan decorations can be accommodated on either side of a ribbon (Figure 1b). The model was built using the proposed rectangular 24-chain fibril structure preferred by Fernandes *et al*. (2011) and Thomas *et al*. (2013). Nevertheless, the steric considerations preventing interactions between acetylated xylan and the hydrophilic cellulose faces hold whether the cellulose microfibrils have hexagonal or square cross sections, and therefore are also true on the (110) and (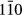) cellulose faces (Figure S1).

### Cleavage of acetylated xylan by xylanases

We investigated whether acetylation of xylan is non-randomly arranged along the xylan backbone. Interpretation of any patterning in acetylation of the xylan backbone is complicated by the presence of GlcA decorations on the xylan. Therefore, we also studied acetylated xylan from the *gux1 gux2* mutant of Arabidopsis stems, which lacks the GlcA decorations (Mortimer *et al*., 2010; Bromley *et al*., 2013). The acetylated xylan from the wild type (WT) and *gux1 gux2* mutants was DMSO-extracted from delignified stem cell walls, and acetylation was then studied by NMR. A gradient-selective ^13^C heteronuclear single-quantum correlation spectroscopy (HSQC) experiment incorporating a long recovery period was recorded for the purpose of quantifying the degree of the different acetylations (Figure S2; Table 1). The percentage of acetylated residues is approximately 50%, similar to that previously reported for glucuronoxylan from WT Arabidopsis (Xiong *et al*., 2013).

**Table 1:** Acetylation of *gux1 gux2* and wild-type (WT) xylan, determined by the integration of H3/C3 peaks in a ^13^C HSQC experiment

	*gux1 gux2* (%)	WT (%)
Non-acetylated X	55.6	46.8
X2	15.3	26.7
X3	26.6	22.2
X23	2.5	4.3

The percentages of xylosyl (X), 2-*O*-acetyl-xylosyl (X2), 3-*O*-acetyl-xylosyl (X3) and 2-*O*-acetyl-3-*O*-acetylxylosyl (X23) residues are shown.

**Figure 2:**
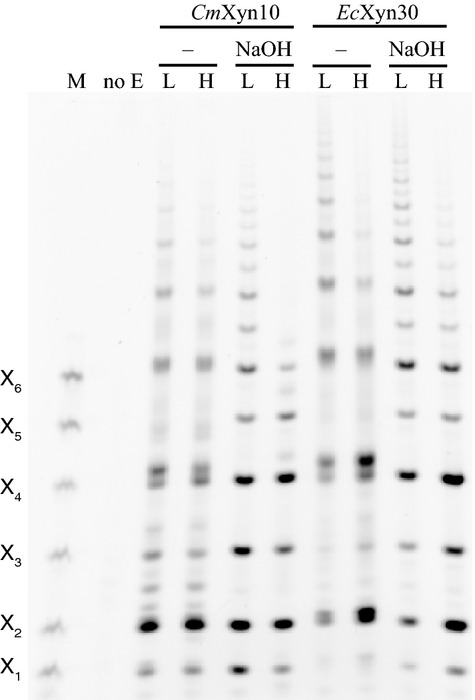
Digestion of *gux1 gux2* mutant Arabidopsis acetylated xylan with *Cm*Xyn10B and *Ec*Xyn30 analysed by PACE. Major oligosaccharides have degrees of polymerization (DPs) of multiples of two xylosyl residues. Digestion was carried out with low (L) or high (H) enzyme loads. After deacetylation with NaOH, the predominantly even DP of products is apparent by comparison with xylo-oligosaccharide markers DP1–DP6 (M). No E, no enzyme digestion acetylxylan control.

The extracted acetylated *gux1 gux2* xylan was partially digested with xylanase 10B from *Cellvibrio mixtus* (*Cm*Xyn10B). Xylanases are impeded by acetylation of the xylan (Biely *et al*., 1986). Family-10 glycoside hydrolases bind decorated substrates and therefore cleave the acetylated xylan non-randomly at specific acetylation arrangements in the backbone. *Cm*Xyn10B cannot accommodate decorations at the −1 subsite, as both the 2- and 3-OH of the xylose face ‘into’ the protein, but can tolerate decorations at the xylose at the +1 subsite, as both carbon 2- and 3-OH of this sugar face outwards into the solvent (Pell *et al*., 2004). With incomplete digestion of the acetylated xylan, Polysaccharide Analysis by Carbohydrate gel Electrophoresis (PACE) showed that a ladder of oligosaccharides of varying length was released by the *Cm*Xyn10B enzyme (Figure 2). The size of these oligosaccharides could be reduced by more extensive digestion of the acetylated xylan, confirming that the longer products were the result of incomplete digestion. The main products migrate close to non-acetylated, even-DP (degree of polymerization) xylan oligosaccharides (Xyl_2_, Xyl_4_ and Xyl_6_), and after deacetylation with NaOH they co-migrated with the ladder. Less abundant odd-DP oligosaccharides were also present.

*Ec*Xyn30 xylanase, previously thought to be specific for glucuronoxylan (Urbanikova *et al*., 2011), was surprisingly also able to cut *gux1 gux2* acetylated xylan lacking GlcA (Figure 2). Again, this enzyme released a ladder of acetylated xylan oligosaccharides, dominated by oligosaccharides differing in length by two sugars. After NaOH deacetylation of the more complete digestion, the main products were clearly DP2, DP4 and DP6. Together, these data suggest the *Cm*Xyn10B and *Ec*Xyn30 xylanases digest non-random sites in the acetylated xylan backbone, and they prefer to cut at sites spaced at an even number of xylosyl residues. This suggests the acetylation is spaced non-randomly, with a pattern associated with an even number xylosyl residues.

### Alternate xylosyl residues are substituted with acetate

**Figure 3:**
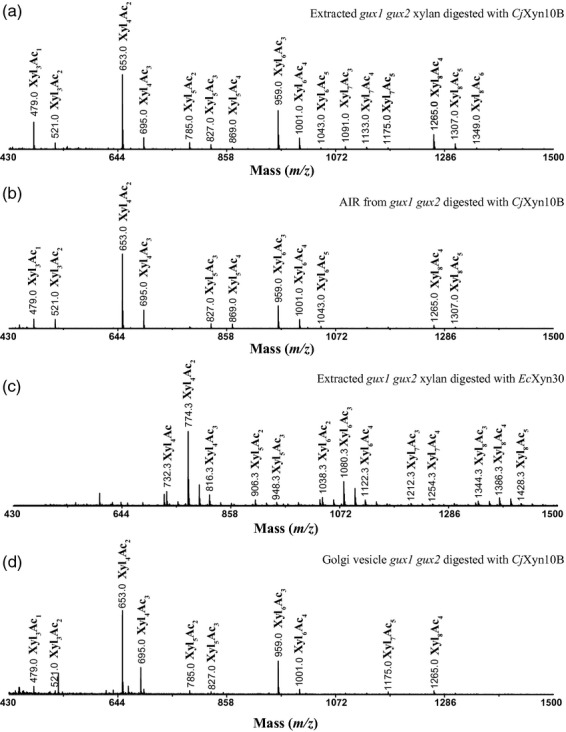
MALDI-ToF-MS of xylanase-digested *gux1 gux2* acetylated xylan. (a) *Cm*Xyn10B digestion of acetylated xylan extracted from delignified *gux1 gux2* stem cell walls. (b) *Cm*Xyn10B digestion of *gux1 gux2* stem cell wall alcohol-insoluble residue. (c) 2-Aminobenzoic acid (2-AA)-labelled *Ec*Xyn30 digestion of acetylated xylan extracted from delignified *gux1 gux2* stem cell walls. (d) *Cm*Xyn10B digestion of acetylated xylan in Golgi membrane vesicles prepared from *gux1 gux2* stems.

To determine the mass and the degree of acetylation of the *Cm*Xyn10B-released oligosaccharides from *gux1 gux2*-acetylated xylan, we used matrix-assisted laser desorption ionization time-of-flight mass spectrometry (MALDI-ToF-MS). The ladder of products revealed higher abundances of xylan DP4, DP6 and DP8 than DP5 and DP7 (Figure 3a), consistent with the PACE results of non-random cleavage of acetylated xylan. The DP4 oligosaccharide most frequently carried two acetyl groups, the DP6 most frequently carried three acetyl groups and the DP8 most frequently carried four acetyl groups. Some oligosaccharides carried additional acetyl groups. The number of acetyl groups is consistent with approximately 50% of residues carrying acetylation (Table 1). To investigate whether this pattern of xylanase-released oligosaccharides was a consequence of delignification and extraction of a fraction of xylan from the wall, xylan was digested directly in alcohol-insoluble cell wall residues. Again, a similar pattern of oligosaccharides was seen (Figure 3b).

**Figure 4:**
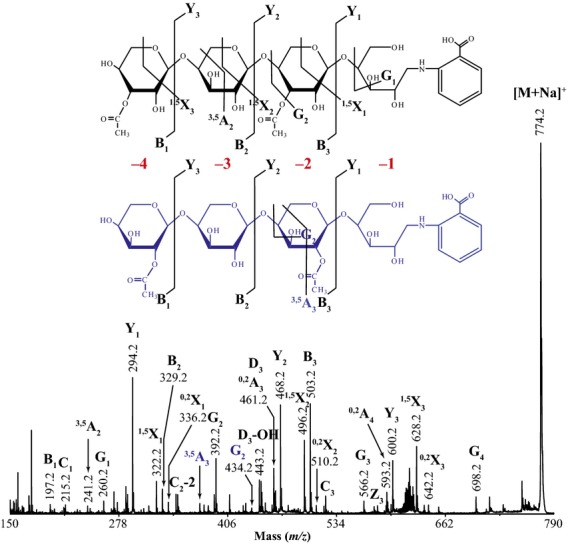
High-energy MALDI-CID MS/MS of the Xyl_4_Ac_2_ oligosaccharide released by *Cm*Xyn10B from *gux1 gux2* acetylated xylan, labelled with 2-AA and separated by HILIC.

To determine the position of the acetyl groups on the acetylated oligosaccharides released by *Cm*Xyn10B, oligosaccharides were labelled with 2-aminobenzoic acid (2-AA), separated by hydrophobic interaction liquid chromatography (HILIC) and analysed by MALDI collision-induced dissociation (CID) MS/MS. Figure 4 shows MALDI-CID MS/MS of Xyl_4_Ac_2_ (*m/z* 774). Interestingly, the acetyl groups were at the −2 and −4 xylose from the reducing end (AcXyl-Xyl-AcXyl-Xyl). Acetate can be accommodated on the 2- or 3-OH of xylose by *Cm*Xyn10B at the +1 site (the non-reducing end of the released oligosaccharide, position −4 of this oligosaccharide), but at the −2 subsite only decoration of 3-OH can be tolerated, as a side chain at 2-OH would clash with the protein (Pell *et al*., 2004; see above). The MS/MS fragmentation indicated some of the acetate groups could be found on the 2-OH and some on the 3-OH of each xylose. This is consistent with the reports that acetyl groups on xylose can migrate between the 2-OH and the 3-OH (Mastihubova and Biely, 2004; Biely *et al*., 2013); therefore, their native position cannot be determined.

High-energy MALDI-CID of oligosaccharide Xyl_4_Ac_3_ *m/z* 816 showed the presence of xylose di-substituted at O2 and O3 with acetate (Figure S3). Interestingly, these were either at position −4 or at position −2. The third acetate was also found on the −4 or −2 xylosyl residue. It is notable that acetate was not found at position −3.

To determine the spacing of acetylation of the oligosaccharides from the *Ec*Xyn30 xylanase digest of acetylated xylan, oligosaccharides labelled with 2-AA were examined by MALDI-ToF-MS (Figure 3c) and high-energy MALDI-CID (Figure S4). The enzyme released Xyl_4_Ac_2_ and Xyl_6_Ac_3_ as the main products. MALDI-CID of the Xyl_4_Ac_2_ products confirmed the even spacing of the acetyl residues at the −2 and −4 non-reducing end xylose, as seen in the *Cm*Xyn10B digest (Figure S4a). MALDI-CID of Xyl_6_Ac_3_ showed acetyl groups predominantly at the −2, −4 and −6 xylose residues (Figure S4b). All the MS studies therefore showed a consistent pattern of acetylation on alternate xylosyl residues.

### NMR indicates that acetylated xylosyl residues are adjacent to non-acetylated residues

**Figure 5:**
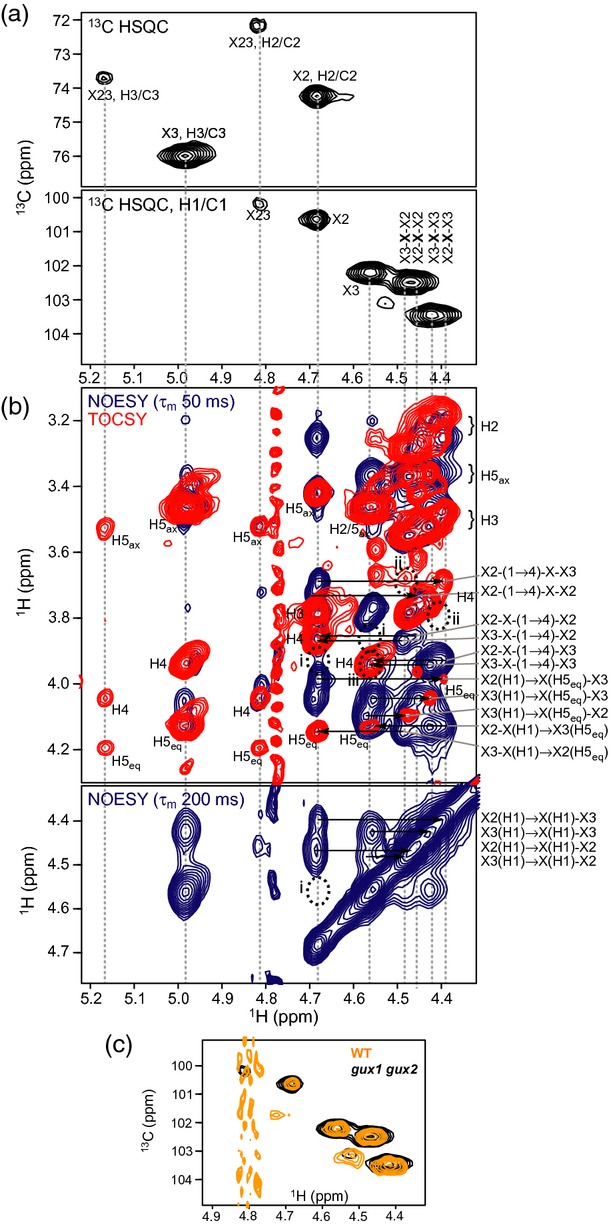
NMR analysis showing relationship of acetylated and non-acetylated xylosyl residues. (a) Two-dimensional ^13^C HSQC spectrum showing the assignment of ^1^H attached to acetylated ^13^C (top panel) and the anomeric region (below). (b) Two-dimensional ^1^H–^1^H NOESY and TOCSY spectra, shown in blue and red, respectively. Detailed analysis of the NOE cross peaks allows the various non-acetylated xylosyl residues to be distinguished. Key NOEs, connecting inter-residue H1 and H4/H5_eq_ in the 50-ms mixing-time experiment, and H1–H1 in the 200-ms mixing-time experiment, are shown by arrows. Dotted circles highlight the absence of NOEs between: (i) X2 and X3; (ii) non-acetylated Xyl with itself, and (iii) X3 and X3. (c) Anomeric region of two-dimensional ^13^C HSQC spectra showing the close similarity of chemical shifts in WT and *gux1 gux2* acetylated xylan.

Intact *gux1 gux2* acetylated xylan was analysed by NMR spectroscopy. Chemical-shift assignments were obtained using ^1^H–^1^H two-dimensional nuclear magnetic resonance spectroscopy (TOCSY) and nuclear Overhauser effect spectroscopy (NOESY) alongside two-dimensional ^13^C HSQC, heteronuclear two-bond correlation (H2BC), HSQC-TOCSY and HSQC-NOESY experiments (Table S1). The 2-, 3- and 2,3-*O*-acetylated Xyl residues (denoted X2, X3 and X23) were readily identified from the characteristic downfield chemical shifts of the H-2 and/or H-3 residues, respectively (Figure 5a). H2BC connections were used to assign intraresidue adjacent ^1^H and ^13^C, combined with TOCSY where overlap led to ambiguities. The extra resolution present in the ^1^H–^1^H TOCSY and NOESY experiments indicated two highly similar environments for each of X2 and X3, which were in each case barely resolvable except where interglycosidic NOE connectivities differed. Interglycosidic NOE and HMBC connections of X2 and X3 invariably led to non-acetylated Xyl residues (Figure 5b).

Four main non-acetylated Xyl species were observed; one partially overlapping pair showed a (1 → 4) connection (i.e. towards the reducing end) to X2, whereas a second pair showed a (1 → 4) connection to X3 (Figure 5b). The species within each pair differed in their non-reducing end connectivity, which was to either X2 or X3. The four non-acetylated Xyl (X) species could thus be identified as the central residue in the four permutations of the triads X2–**X**–X2, X2–**X**–X3, X3–**X**–X2 and X3–**X**–X3. Following from this, the two closely overlapping signals seen for each of X2 and X3 reflect a long-range sensitivity to the nature of the sugar two residues towards the reducing end, which carries either the same or differing acetylation. A low population of X23 was observed, and although some resonances of a non-acetylated Xyl connected at the non-reducing end could also be assigned, the low intensity and peak overlap precluded further assignment of this species.

No NOE cross-peaks were detected between X2 and X3 (Figure 5b, see dotted circles marked ‘i’). Additionally, no NOE cross-peaks were detected between non-acetylated Xyl residues (see dotted circles marked ‘ii’). Although it is not impossible that adjacent acetylated or adjacent non-acetylated species exist but overlap to the degree that the interglycosidic connection cannot be distinguished from internal NOE connections, no intense ‘internal’ H1–H4 NOEs were seen at shorter mixing times (see for example the dotted circle marked ‘iii’ for X3), indicating that these species, if present, were relatively low in number (the 1 → 4 NOE would be strong in the presence of a glycosidic linkage). From this we infer that AcXyl-AcXyl and nonAcXyl-nonAcXyl are not present in significant quantities in acetylated *gux1 gux2* xylan.

In summary, the NMR data strongly indicate that in *gux1 gux2* xylan acetylated residues are largely adjacent to non-acetylated residues, and vice versa, i.e. acetylated xylan is predominantly composed of alternating Xyl and AcXyl units.

### Acetylation of alternate residues is seen in xylan from extracted Golgi vesicles and from wild-type Arabidopsis stems

The patterning of the acetylation of xylan in the *gux1 gux2* plants might arise during biosynthesis in the Golgi, as a result of the specific action of acetyltransferases. Alternatively, the pattern might arise by the removal of specific acetates by esterases in the cell wall. To investigate this, we extracted Golgi-enriched membranes from Arabidopsis inflorescence stems active in xylan synthesis. Xylan in the membranes was digested by *Cm*Xyn10B and the oligosaccharides studied by MALDI-ToF-MS. As seen in Figure 3(d), the xylan in the Golgi apparatus has similar acetylation patterns as the xylan in the cell wall.

**Figure 6:**
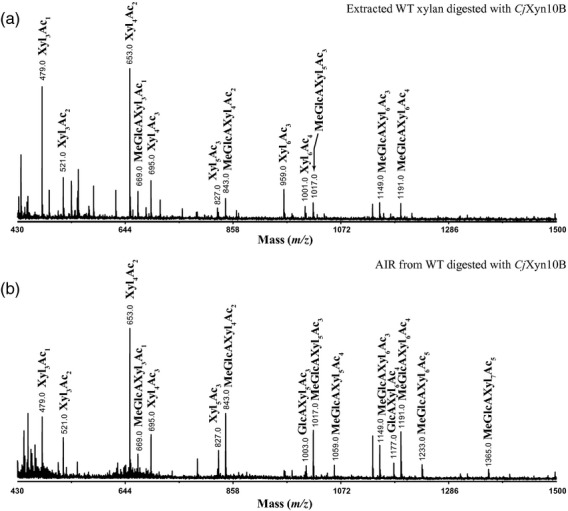
MALDI-ToF-MS of xylanase-digested wild-type (WT) acetylated xylan. (a) *Cm*Xyn10B digest of acetylated xylan extracted from delignified WT stem cell walls. (b) *Cm*Xyn10B digestion of WT stem cell wall alcohol-insoluble residue.

The WT Arabidopsis xylan has GlcA decorations in addition to acetylation. To determine whether xylan in WT plants also has patterned xylan acetylation, we digested both extracted acetylated xylan and intact cell wall material with *Cm*Xyn10B. As seen in Figure 6, acetylated oligosaccharides substituted by GlcA (with or without 4-*O*-Me) were detected by MALDI-ToF-MS. The oligosaccharides without GlcA substitution were dominated by Xyl_4_Ac_2_. MALDI-CID of the 2-AA-labeled Xyl_4_Ac_2_ oligosaccharide confirmed the locations of acetate at the −4 and −2 Xyl, as seen in the *gux1 gux2* mutants (Figure S5). Similar MS/MS fragmentation results have been very recently shown for MeGlcAXyl_4_Ac_2_ (Chong *et al*., 2014).

The NMR chemical shifts of WT xylan were identical to those of the *gux1 gux2* mutant, with the exception of the new peaks arising from GlcA substitutions, implying the same pattern of acetylation. The anomeric regions of the ^13^C HSQC are shown overlain in Figure 5(c).

### Molecular dynamics simulations of naked and substituted xylans on cellulose fibrils

As the acetylation and glucuronosylation (Bromley *et al*., 2013) of a substantial proportion of the xylan follows an evenly spaced pattern, we conducted molecular dynamics (MD) simulations to determine whether two-fold helical screw (2_1_-fold) xylan would stably interact with a Iβ microfibril. The rectangular cross section 24 chain cellulose microfibril has (010) and (020) hydrophilic and (100) and (200) hydrophobic surfaces (Figure S6) (Fernandes *et al*., 2011; Gomes and Skaf, 2012). The simulations showed that unsubstituted 2_1_-fold xylan has the ability to interact with either hydrophilic or hydrophobic surfaces of cellulose, with interaction potential energies ranging from about −100 to −150 kcal mol^−1^ for a stretch of xylan with DP10 (Tables S2–S5): that is, from −10 to −15 kcal mol^−1^ per xylosyl residue. Evenly spaced substitution has no statistically significant effect on the interaction energy between different xylans and cellulose on surfaces (010) or (020) (Table S2). On hydrophobic surfaces (100) and (200), adsorption of acetylxylan may be slightly less stable, and glucuronoxylan may be slightly more stable, when compared with unsubstituted xylan. Nevertheless, on all surfaces, with all three different xylans, stable adsorption complexes are formed. Hence, evenly substituted, 2_1_-fold xylan ribbons are energetically feasible candidates for our adsorption model in which xylan molecules adsorb to cellulose surfaces at crystallographic surface vacancies.

**Figure 7:**
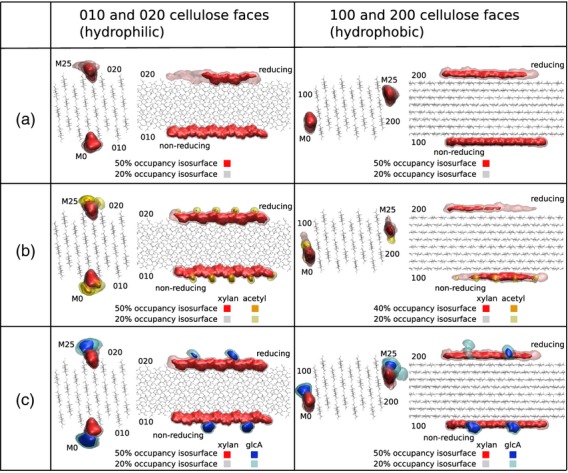
Molecular dynamics simulation of xylan (a, unsubstituted; b, acetylated; c, glucuronosylated) interacting with hydrophilic (010) and (020), or hydrophobic (100) and (200), surfaces of cellulose. Occupancy-level isosurfaces are shown. Dark- and light-coloured isosurfaces represent spatial regions where substituted xylan is present 40–50% and 20% of the simulation time, respectively.

We monitored the binding of xylan on cellulose during the course of the simulations. The results for all simulated systems show that, on all surfaces, xylans (unsubstituted, acetylxylan and glucuronoxylan) spend much of the simulation time near the adsorption site (Figure 7a–c, respectively). On the (010) and (100) faces, xylan molecules do not leave their adsorption sites. On the (200) face, the xylan chains behave very similarly, except for an augmented mobility, especially towards its reducing end. On face (020), the xylan molecules exhibit higher mobility towards the non-reducing end, and stretches of the xylan molecules transiently desorb from this surface. The higher mobility resulting from acetylation on the (100) and (200) faces is consistent with the slightly lower interaction energies of acetylxylan with the hydrophobic surfaces (Table S2), as compared with unsubstituted xylan. Faces (010) and (020) are both hydrophilic, and marked differences in xylan adsorption properties on these surfaces are not expected. The differences between xylan adsorption on the (020) and (010) faces seen in Figure 7(a) are the result of a slight tilt of the plane of origin chains in the cellulose microfibril observed during the simulations (Figure S7). It is unclear whether this effect arises from shortcomings of the force field or represents an actual behavioural difference to be expected between origin and centre chains.

### Influence of substitutions on docked xylan interaction with water

The MD simulations show that xylan acetylation has little effect on its interaction energy with water, whereas GlcA residues on xylan strongly stabilize its interaction with water by about −400 kcal mol^−1^ (Table S3). This energy difference largely arises from the electrostatic contribution. The strong interaction with water has little or no effect on the interaction energy of glucuronoxylan with cellulose, compared with unsubstituted xylan (Table S4). Similarly, acetyl moieties in evenly spaced acetylated xylan also do not affect the interaction energy of xylan with the (010) cellulose surface, but in this case acetyl moieties alone confer little stabilization to the interaction with surrounding water (Table S5). The results in Tables S4 and S5 thus indicate that the evenly spaced chemical functionalization of xylan allows the tuning of interactions between xylan and the surrounding medium, without compromising the interaction of xylan with cellulose hydrophilic surfaces.

### Xylan exhibits a 2_1_-fold helical screw when adsorbed onto cellulose surfaces, and a 3_1_-fold helical screw in water

**Figure 8:**
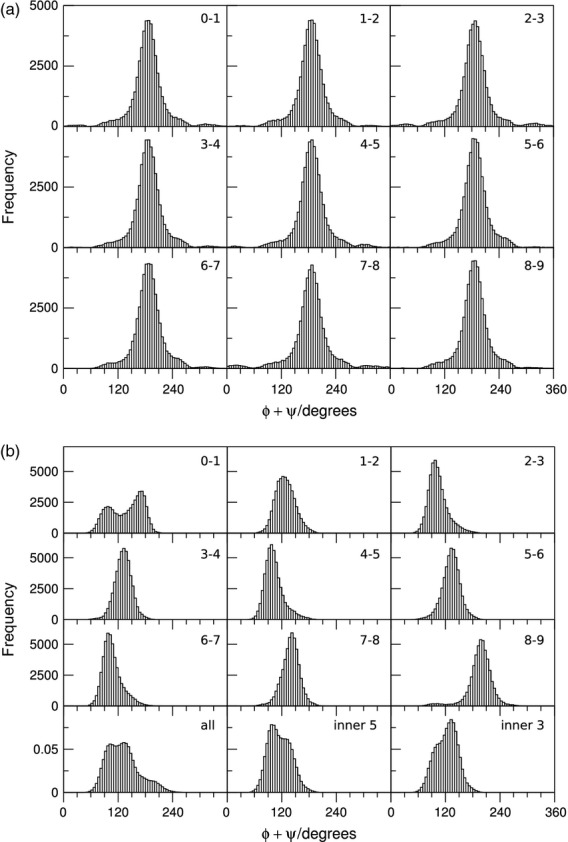
Xylan in 3_1_- and 2_1_-fold screw conformations. Histograms showing the distribution of glycosidic dihedral angles Φ + Ψ between adjacent xylose residues of unsubstituted xylan of DP10. Numbers refer to xylose residues. (a) Xylan in water: Φ + Ψ ∼ 190° in water indicates threefold (3_1_) helical conformation. (b) xylan on cellulose face 010. Φ + Ψ ∼ 120° indicates twofold (2_1_) conformation.

The sum of dihedrals Φ and Ψ at a particular glycosidic oxygen is indicative of local glycan conformation at that oxygen (Mazeau *et al*., 2005; French and Johnson, 2009). In particular, for β-(1-4)-linked glycans, if the sum Φ + Ψ equals 120° at all glycosidic oxygens, such glycan displays a 2_1_ helical conformation along the whole polymeric chain. The glycan assumes threefold helical, 3_1_, conformation when Φ + Ψ equals 50° (right handed) or 190° (left handed) at all glycosidic oxygens. Results of MD simulations of a free xylan molecule in water, shown in Figure 8(a), indicate that in water the distribution of Φ + Ψ is centred around 190°, consistent with a 3_1_-fold screw. For xylan adsorbed to surface (010), in contrast, the Φ + Ψ distribution is centred at 120° (Figure 8b), indicative of twofold helical conformations. As xylan on face (010) does not leave its adsorption groove during simulations (Figure 7a), the 2_1_ conformations correspond to adsorbed xylan. Xylan adsorbed to the other crystallite surfaces also showed a population with a Φ + Ψ distribution centred at 120°, particularly for the internal xylosyl residues (Figure S8). On the (020) and (200) faces a population with a Φ + Ψ distribution centred at 190° was also observed.

**Figure 9:**
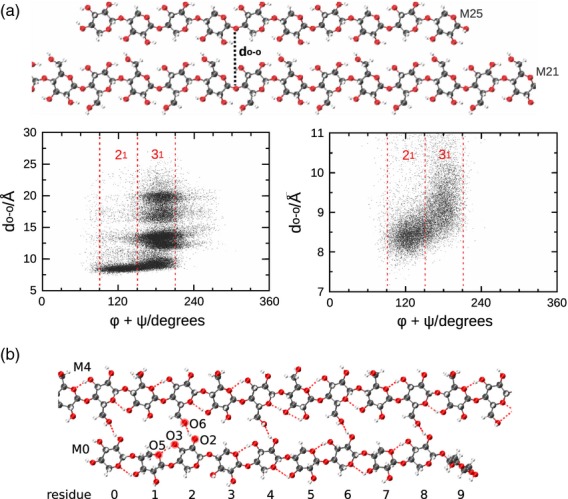
Neighboring xylan and glucan chains for xylan (DP10) adsorbed on the hydrophilic faces of cellulose. (a) Scattered plot of the interchain distance against the sum of dihedrals Φ + Ψ, showing that when the chains are close to each other (xylan adsorbed onto cellulose) only xylosyl twofold screw, 2_1_, conformations occur, whereas when the chains are farther apart, xylan assumes a threefold screw, 3_1_, conformation. (b) The predominant xylan–glucan hydrogen-bonding mode has xylosyl O2 as the proton donor and glucosyl O6 as the proton acceptor.

On surface (020), xylosyl residues transiently desorb (Figure 7a), making incursions into the water bulk. This behaviour is reflected in the scatter plot of the xylan Φ + Ψ sum against the xylan–glucan interchain separation, *d*_O–O_, shown in Figure 9(a). For small separations, the sum of dihedrals is centred at 120°, whereas for higher values of *d*_O–O_, Φ + Ψ is centred at 190°. These results indicate that local 2_1_ conformations correspond to adsorbed xylosyl residues, whereas 3_1_ conformations correspond to stretches of desorbed xylan interacting with water.

### Evenly spaced substitution does not disrupt the hydrogen-bonding network between xylan and hydrophilic cellulose surfaces

When adsorbed to cellulose hydrophilic surfaces (010) and (020), xylan establishes several hydrogen bonds to the cellulose molecule directly above or below it. The overall trend for xylan molecule M0, on face (010), is that alternate residues (residues 0, 2, 4, 6 and 8) hydrogen-bond to cellulose chain M4. For the intervening non-hydrogen-bonded xylosyl residues, the O2 and O3 major candidates for hydrogen-bonding are pointed away from the cellulose fibril, as shown in Figure 9(b). The trend is similar for xylan M25 on face (020).

Hydrogen-bonding statistics computed from the MD trajectories (Tables S6 and S7) reveal that the predominant xylan–cellulose hydrogen-bond mode takes place with xylosyl O2 as proton donors and glucosyl O6 as acceptors. At times, this relationship may be inverted, with xylosyl O2 acting as acceptors and glucosyl O6 as donors. In the very short time intervals when cellulose O6 is not bonding to xylan O2, it establishes hydrogen bonds with xylan O3 and, at times, cellulose O6 may interact with both xylan O2 and O3 simultaneously. From these observations we conclude that cellulose O6 is paramount for hydrogen bonding between cellulose and xylan. The pattern of even substitution on xylan preserves the original hydrogen-bond network between xylan and hydrophilic cellulose surfaces. As the substituted hydroxyls in xylan point outwards from the cellulose crystallite, hydroxyls involved in hydrogen bonding are not disturbed by the even substitution pattern.

## Discussion

We have demonstrated that xylan acetylation predominantly occurs at alternate xylosyl residues. First, exploiting the preference of the xylanase for an undecorated –1 residue and tolerance of decoration at the +1 residue, *Cm*Xyn10B digestion of acetylated *gux1 gux2* stem xylan resulted in a pattern of even-length oligosaccharide products (Figure 2). The bands were not as sharp as normal PACE separations, which may be explained by the mobility of the acetyl groups, shifting between O2 and O3 positions of the xylose residue (Mastihubova and Biely, 2004; Biely *et al*., 2013). After deacetylation with alkali, the oligosaccharides were more clearly dominated by even DP. Second, MALDI-ToF-MS of the xylanase products confirmed the preferred even number of xylosyl residues. Third, high-energy MALDI-CID MS/MS indicated that acetylation of the products was largely at alternate xylosyl residues in the products. Fourth, the same preferences and products were found with a second enzyme, *Ec*Xyn30 glucuronoxylanase. Fifth, NMR indicates that the acetylated xylosyl residues are largely adjacent to non-acetylated residues. A random arrangement can be excluded. The result also indicates that the enzymes digest a representative fraction of acetylated xylan.

It was interesting that acetylated xylan lacking GlcA (from the *gux1 gux2* mutant) can also by digested by *Ec*Xyn30. This activity has not been previously reported, and CAZy GH30 family activity was thought to be restricted to [Me]GlcA substituted xylan (Hurlbert and Preston, 2001; St John *et al*., 2011; Urbanikova *et al*., 2011). The GlcA side chain on the O2 of xylose at −2 has been proposed to be essential for cleavage of the xylan backbone by GH30 glucuronoxylanases, and the structures of two such enzymes in complex with decorated oligosaccharides reveals a discrete [Me]GlcA binding site (St John *et al*., 2011; Urbanikova *et al*., 2011). Although this site is optimized for [Me]GlcA binding, accommodation of an acetyl group is not precluded. The cleavage of acetylated xylan indicates that GlcA is not essential, and suggests that acetyl groups on xylan may play a role in GH30 activity in cell wall degradation by microbes.

To investigate whether the xylan is synthesized with the acetylation pattern or whether it arises in the cell wall, perhaps by specific deacetylation, we analysed xylan from extracted Golgi vesicles. The acetylation pattern was similar in the nascent xylan, indicating it occurs already at the point of biosynthesis in the Golgi apparatus. The xylan backbone synthesis requires IRX9, IRX10 and IRX14, although it is not yet clear what their exact roles are. The acetylation requires the putative acetyltransferase protein Eskimo/TBL29 and other uncharacterized components. A specific interaction between the acetyltransferases and the xylan backbone synthesis enzymes may be required to generate the pattern, such that adjacent residues do not receive acetylation.

We present evidence here that the majority of *gux1 gux2* xylan has a pattern of acetylation of alternate xylosyl residues. We could also detect similar patterning of acetylation in wild-type glucuronoxylan; however, we recently showed that there are two distinct structural domains in xylan molecules (Bromley *et al*., 2013): the major domain has evenly spaced GlcA, whereas the minor domain has more tightly spaced GlcA without any preference for even spacing. We have not yet investigated whether the patterning of acetate is similar in the two domains; however, the even patterning of acetylation is likely to be present in the major domain, based on the quantity of the oligosaccharides released by the hydrolases and the NMR study of WT xylan. This proposal would be consistent with the proposed function of the patterning, namely to allow the twofold helical screw xylan to fold as a ribbon, with acetate and GlcA decorations facing in the same direction.

**Figure 10:**
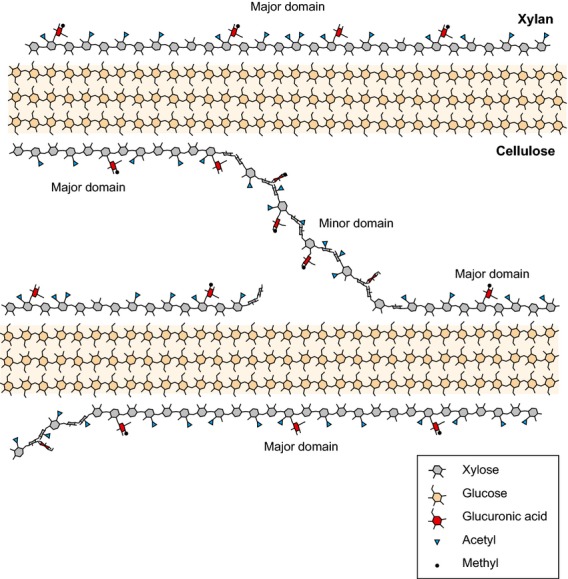
Hypothetical model of cellulose–xylan interactions in the secondary cell walls of dicots. A single plane of a cellulose crystallite with a partial xylan shell in a 2_1_-fold screw is shown. The major domain is accommodated on vacancies on the hydrophilic surface. The minor domain cannot bind to the hydrophilic surface as a 2_1_-fold screw.

The substitution of xylan by sugars and acetate is not only a matter of type and quantity, but also of arrangement. The xylan substituted at even-numbered xylose residues, when folded into a 2_1_-fold helical screw, would allow a three-dimensional structure with flat interface on one side, available for putative interactions with cellulose, and a substitution-rich interface on the other side. Our MD simulations show that the formation of stable adsorption complexes between xylan adopting 2_1_-fold helical screw and crystalline cellulose Iβ is feasible. Second, the simulations show that an even substitution pattern on xylan is a prerequisite to interact with cellulose surfaces at hydrophilic sites. We propose a model of interaction of xylan with cellulose in the secondary cell wall of dicots, presented in Figure 10.

Vacancies are present on hydrophilic cellulose faces, where additional cellulose chains could have been present in larger crystallites. Owing to the structural similarities between Xyl and Glc, they are obvious adsorption sites for xylan chains. Previous work (Hanus and Mazeau, 2006; Zhang *et al*., 2011; Mazeau and Charlier, 2012) has investigated the dynamics of glycan adsorption onto cellulose surfaces but, as far as we know, none has explicitly considered cellulose chain vacancies on crystalline surfaces as preferred adsorption sites for xylan molecules. Here, we provide MD evidence for the feasibility of such interactions if the xylan decorations do not obstruct such an interaction. We have now also shown that the major xylan substitutions are arranged such that xylan can align with the vacancies on cellulose microfibril hydrophilic surfaces. Therefore, we predict that the xylan–cellulose complexes proposed here are very likely to be present in plant cell walls.

Minor domains of xylan possess GlcA in a non-evenly spaced pattern (Bromley *et al*., 2013). It is possible that this domain may not possess alternately acetylated residues, as we found some odd-length oligosaccharides by enzyme hydrolysis. Although this would prevent interaction with the hydrophilic surfaces, using MD simulations we found that such xylan, in the 2_1_-fold helical screw, may interact with hydrophobic faces of cellulose. Thus, the minor domain may interact with different faces of cellulose. It is also possible that this domain can serve as a linker or spacer between cellulose chains. Defective acetylation of plant xylan may not only lead to its aggregation but, according to our model, may also alter interactions of xylan with cellulose. Such an impact on cell wall structural stability could explain, at least in part, the collapse of secondary cell wall xylem vessels in *rwa* and *tbl29* mutants.

Xylan on the hydrophilic surface of cellulose may perform a function of preventing cellulose crystallites from aggregating. Xylan should be more hydrophobic than cellulose because it has hydrogen atoms where cellulose has hydroxymethyl groups. Substitution of xylan -OH groups by acetylation at O2 or O3 positions increases its hydrophobicity even further. According to the simulations and to our model, the increased hydrophobicity provided by acetylation occurs only on the outward-facing side of an evenly acetylated, 2_1_-fold xylan chain. Therefore, it is possible that xylan may act as a compatibilizer (Utracki, 2002) between the hydrophilic surfaces of cellulose and the hydrophobic lignin matrix in cell walls by decreasing the interfacial tension between these two components of the wall, and thus enhancing the adhesion of one component onto the other. Interestingly, Reis and Vian (2004) proposed a model in which short regions of xylan, adsorbed onto cellulose, present GlcA outwards from cellulose, providing the putative lignin binding sites to solidify the structure. Chemically different substituents, such as GlcA, Ara and acetate, may lead to a more general biological strategy for cellulose modification by means of surface coating with xylan molecules.

## Experimental Procedures

### Plant material and growth conditions

All Arabidopsis plants were of the Col-0 ecotype. The *gux1-2* *gux2-1* double T-DNA insertion line mutant (SALK_046841/GABI_722F09) has been described previously (Bromley *et al*., 2013). Arabidopsis seeds were surface-sterilized and stratified in darkness for 48 h at 4°C, then sown onto soil and grown in a growth room (20°C, 100 μmol m^−2^ s^−1^, 16-h light/8-h dark, 60% humidity).

### Cell wall preparation and extraction of acetylated xylan

Alcohol-insoluble residue (AIR) was prepared as described by Bromley *et al*. (2013). Before extraction, AIR was depectinated using 0.5% ammonium oxalate. Acetylated xylan was extracted according to the procedure described in Goncalves *et al*. (2008). Briefly, the AIR was delignified using 11% peracetic acid for 30 min at 85°C. The water-washed holocellulose was treated with DMSO for 48 h in total (2 × 24 h) at 60°C, to extract acetylated hemicelluloses. After extraction, xylan was recovered from DMSO using PD-10 desalting columns (GE Healthcare, http://www.gehealthcare.com).

### Preparation of Golgi vesicles

Approximately 20 g of stems from 4–week-old plants were harvested and homogenized in homogenization buffer (250 mm sucrose, 10 mm HEPES, pH 7.5, 1 mm EDTA, 1 mm DTT). The homogenate was spun at 2200 ***g*** to pellet the unbroken cells, cell walls, nuclei, and most of the plastids and mitochondria. The supernatant was centrifuged in an SW28 rotor at 100 000 ***g*** for 2 h at 4°C onto an 18% iodixanol cushion. The concentrated microsomes were collected from the interface and spun for 30 min at 4°C in a rotor at 100 000 ***g***. The pellets were used to extract alcohol-insoluble residues, as described above.

### Enzyme hydrolysis and visualization of oligosaccharides with polysaccharide analysis by carbohydrate gel electrophoresis (PACE)

Acetylated xylan, or holocellulose (depectinated and delignified AIR), was hydrolysed in 0.1 m ammonium acetate buffer (*Cm*Xyn10B*,* pH 5.5; *Ec*Xyn30 pH 6.0) for 24 h at 21°C before boiling for 30 min to heat inactive enzymes. Enzymes used in this study were: *Cm*Xyn10B at 0.08 μm (low concentration) and 0.8 μm (high), and *Ec*Xyn30 at 0.14 μlm (low) and 1.4 μm (high) (Pell *et al*., 2004; Urbanikova *et al*., 2011). The labelling of digestion products and standards, gel running and visualization were carried as described in Bromley *et al*. (2013).

### NMR

NMR spectra were recorded at 298 K with a Bruker AVANCE III spectrometer operating at 600 MHz equipped with a TCI CryoProbe. Two-dimensional ^1^H–^1^H TOCSY, NOESY, ^13^C HSQC, H2BC, HMBC, HSQC-TOCSY and HSQC-NOESY experiments were performed, using established methods (Cavanagh *et al*., 1996; Nyberg *et al*., 2005). The TOCSY mixing time was 70 ms, and NOESY experiments were recorded at mixing times of 50, 100 and 200 ms. Chemical shifts were measured relative to internal acetone (δH = 2.225, δC = 31.07 ppm). Data were processed using the azara 2.8 suite of programs (copyright 1993–2014, Wayne Boucher and Department of Biochemistry, University of Cambridge), and chemical-shift assignment and peak integrations were performed using analysis 2.4 (Vranken *et al*., 2005).

### Preparation of oligosaccharides from cell walls for mass spectrometry

For the preparation of native oligosaccharides, 100 μg of AIR were resuspended in 100 μl of 50 mm ammonium acetate, pH 5.5, vigorously vortexed and heated at 95°C for 10 min. Hydrolases were added and digested overnight (16 h) at 37°C, with shaking at 300 rpm. Dowex 50WX8 cation exchange resin beads in water (10%) were added to desalt and remove enzymes.

### Reductive amination of acetylated xylooligosaccharides and purification

The acetylated xylooligosaccharides were reductively aminated with 2-AA (Sigma-Aldrich, http://www.sigmaaldrich.com) using optimized labelling conditions, described previously (Maslen *et al*., 2007), and were purified from the reductive amination reagents using a Glyko Clean S cartridge (Prozyme, http://www.prozyme.com), as described by Tryfona *et al*. (Tryfona and Stephens, 2010).

### MALDI-ToF-MS/MS

Native or reductively aminated samples were analysed by MALDI-ToF-MS/MS (4700 Proteomics Analyser; Applied Biosystems, http://www.appliedbiosystems.com), as previously described (Maslen *et al*., 2007), using 2,5-dihydroxybenzoic acid (2,5-DHB) matrix (10 mg ml^−1^ dissolved in 50% MeOH). The above tandem mass spectrometer uses a 200-Hz frequency triple Nd-YAG laser operating with a wavelength of 355 nm. High-energy MALDI-CID spectra were acquired with an average of 10 000 laser shots/spectrum, using a high collision energy (1 kV). The oligosaccharide ions were allowed to collide in the CID cell with argon at a pressure of 2 × 10^−6^ Torr (0.27 mPa).

### HILIC-MALDI-ToF-MS/MS

Capillary HILIC was carried out with an amide-80 column, as previously described (Anders *et al*., 2012). For HILIC-MALDI-ToF/ToF tandem mass spectrometry a Probot sample fraction system (Dionex, http://www.thermoscientific.com/dionex) was employed for automated spotting of the HPLC eluent onto a MALDI target at 20-s intervals.

### Molecular dynamics

The 24-chain square cellulose fibrils with adsorbed xylans were built with cellulose builder (Gomes and Skaf, 2012). Periodic boundary conditions were applied so that each cellulose chain in the crystallite is covalently bonded to its periodic images at both ends. Cellulose chains have DP20, and xylan (naked and substituted) have DP10. The simulation boxes were filled by approximately 13 200 explicit TIP3P water molecules (Jorgensen *et al*., 1983) using Packmol (Martinez *et al*., 2009), so that the carbohydrates are surrounded by a water layer of at least 12 Å thick. The CHARMM force field was used for the carbohydrates (Guvench *et al*., 2008, 2009). All simulations were performed using namd (Phillips *et al*., 2005). Energy was first minimized using up to 4000 steps of the conjugate gradient method. The systems where thermalized during a 2-ns simulation in the NPT ensemble at 1 bar and 300 K, using the Nosé–Hoover barostat and the Langevin thermostat, as implemented in NAMD. Average volumes from the last nanosecond of the equilibration runs were subsequently used to restart simulations in the NVT ensemble. The velocity Verlet integrator was used with a time step of 2 fs. Short-range interactions were subjected to a 12-Å cut-off, with a 10–12 Å switching function, and particle mesh Ewald sums were used for the electrostatic interactions in namd. For each system, two independent simulations of 50 ns were performed. Covalent bonds involving hydrogen atoms were kept at fixed bond lengths with shake (Ryckaert *et al*., 1977). All data analyses were carried out using either vmd (Humphrey *et al*., 1996) and/or in-house scripts and programs.

## SUPPORTING INFORMATION

**Figure S1.** Model of acetylated xylan interacting with cellulose with hexagonal cross section.

**Figure S2.** H3/C3 regions of a two-dimensional ^13^C HSQC spectrum.

**Figure S3.** MALDI-CID of Xyl_4_Ac_3_ released by *Cm*Xyn10B from acetylated *gux1 gux2* stem xylan, labelled with 2-AA and separated by HILIC.

**Figure S4.** MALDI-CID on the Xyl_4_Ac_2_ released by *Ec*Xyn30 from acetylated *gux1 gux2* stem xylan.

**Figure S5.** MALDI-CID of Xyl_4_Ac_2_ released by *Cm*Xyn10B digestion of acetylated xylan from wild-type Arabidopsis stems.

**Figure S6.** Numbering of individual molecules used in the simulations.

**Figure S7.** Two MD snapshots showing the slight tilting of the plane of origin chains observed during simulations.

**Figure S8.** Histograms showing the distribution of glycosidic dihedral angles Φ + Ψ between adjacent xylose residues of unsubstituted xylan DP10.

**Table S1.** ^1^H and ^13^C NMR assignments of acetylated *gux1 gux2* xylan at 25°C in D_2_O.

**Table S2.** Interaction energies between xylans and cellulose.

**Table S3.** Interaction energies between xylans and water.

**Table S4.** Glucuronoxylan–cellulose and glucuronoxylan–water interaction energies.

**Table S5.** Acetylxylan–cellulose and acetylxylan–water interaction energies.

**Table S6.** Xylan–cellulose hydrogen bonding statistics for xylans adsorbed on the (010) face of cellulose.

**Table S7.** Xylan–cellulose hydrogen bonding statistics for xylans adsorbed on the (020) face of cellulose.
