# Supplementary figures and images for: The pattern of xylan acetylation suggests xylan may interact with cellulose microfibrils as a twofold helical screw in the secondary plant cell wall of Arabidopsis thaliana
Source: Plant J. 2014 Jun 6;79(3):492–506. doi: 10.1111/tpj.12575 (PMC4140553; doi:10.1111/tpj.12575)

acetyl  
xylan  
cellulose

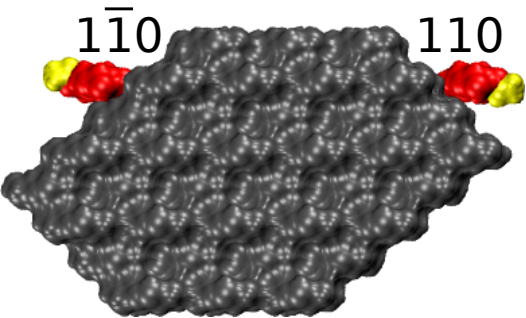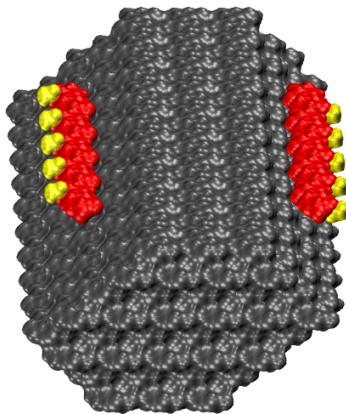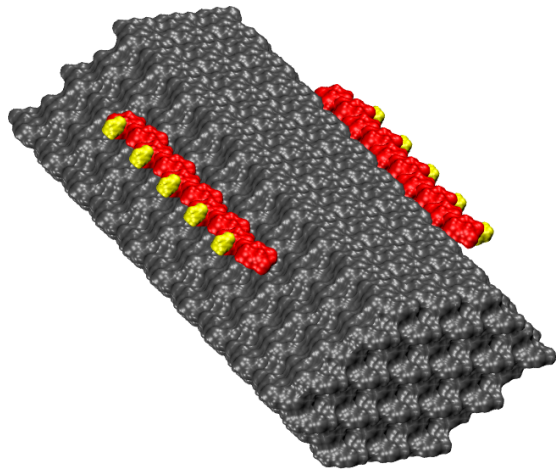

Supplement: Supplementary file 1 — Figure S1. Model of acetylated xylan interacting with cellulose with hexagonal cross section. [file tpj0079-0492-SD1.pdf]

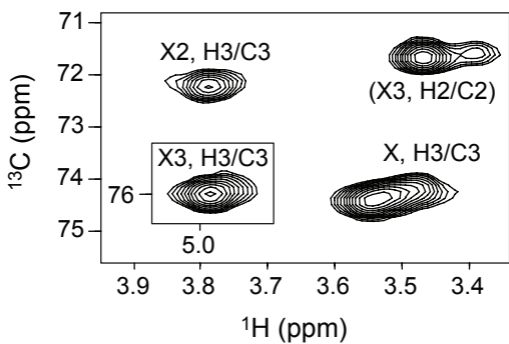

Supplement: Supplementary file 2 — Figure S2. H3/C3 regions of a two-dimensional 13C HSQC spectrum. [file tpj0079-0492-SD2.pdf]

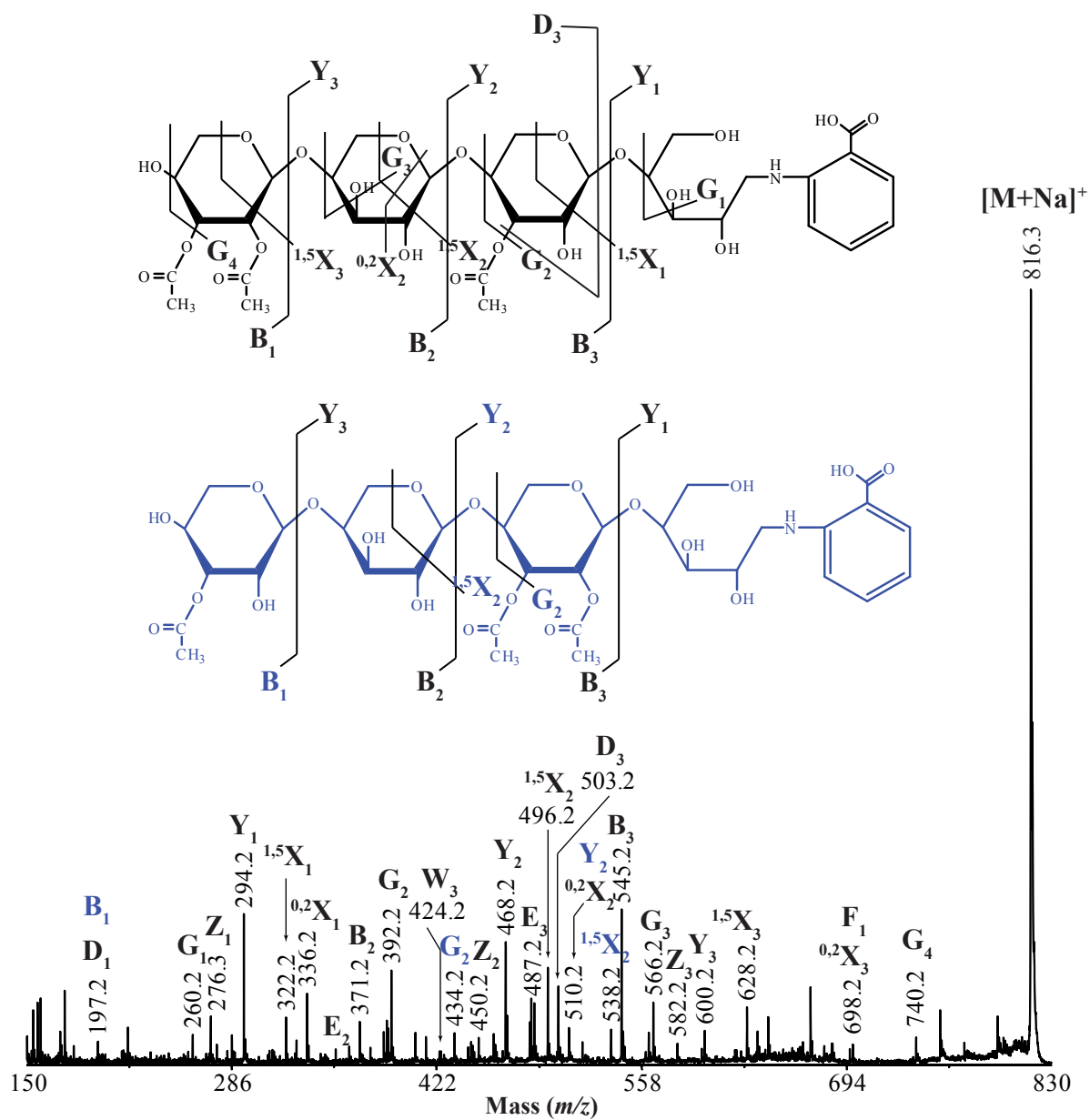

Supplement: Supplementary file 3 — Figure S3. MALDI-CID of Xyl4Ac3 released by CmXyn10B from acetylated gux1 gux2 stem xylan, labelled with 2-AA and separated by HILIC. [file tpj0079-0492-SD3.pdf]

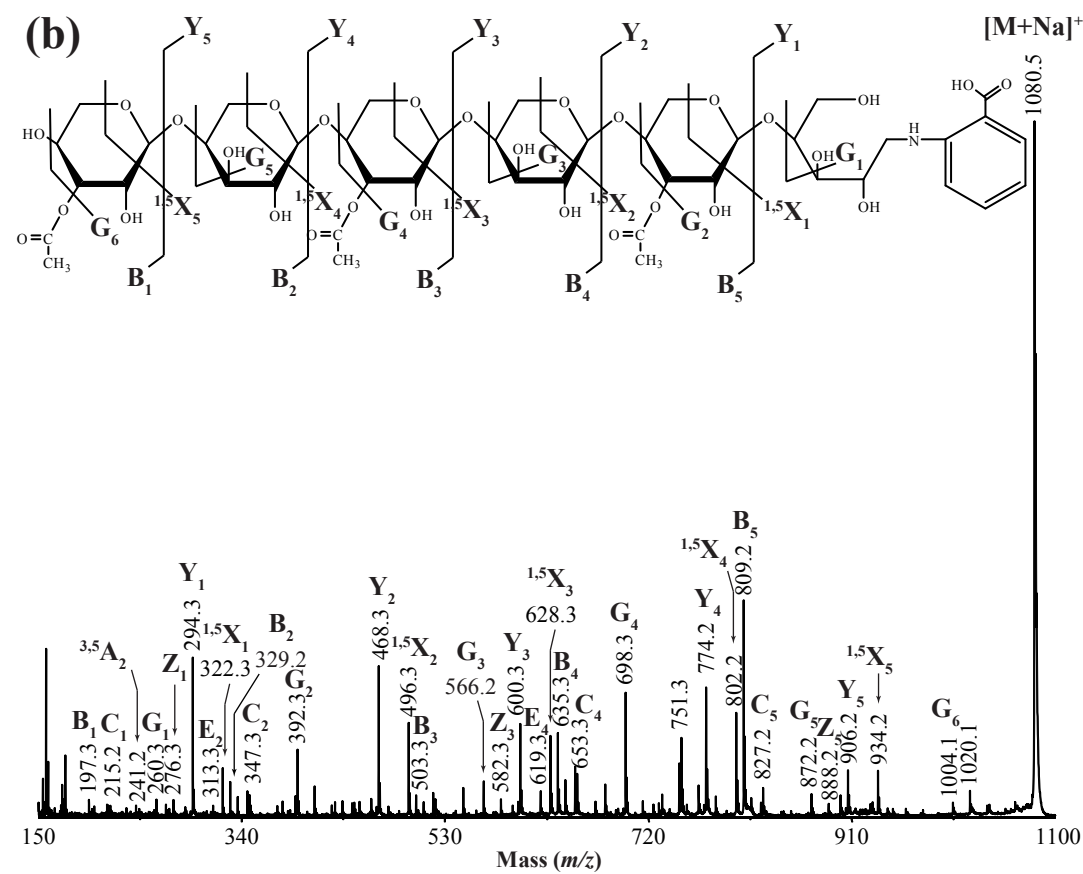

Supplement: Supplementary file 4 — Figure S4. MALDI-CID on the Xyl4Ac2 released by EcXyn30 from acetylated gux1 gux2 stem xylan. [file tpj0079-0492-SD4.pdf]

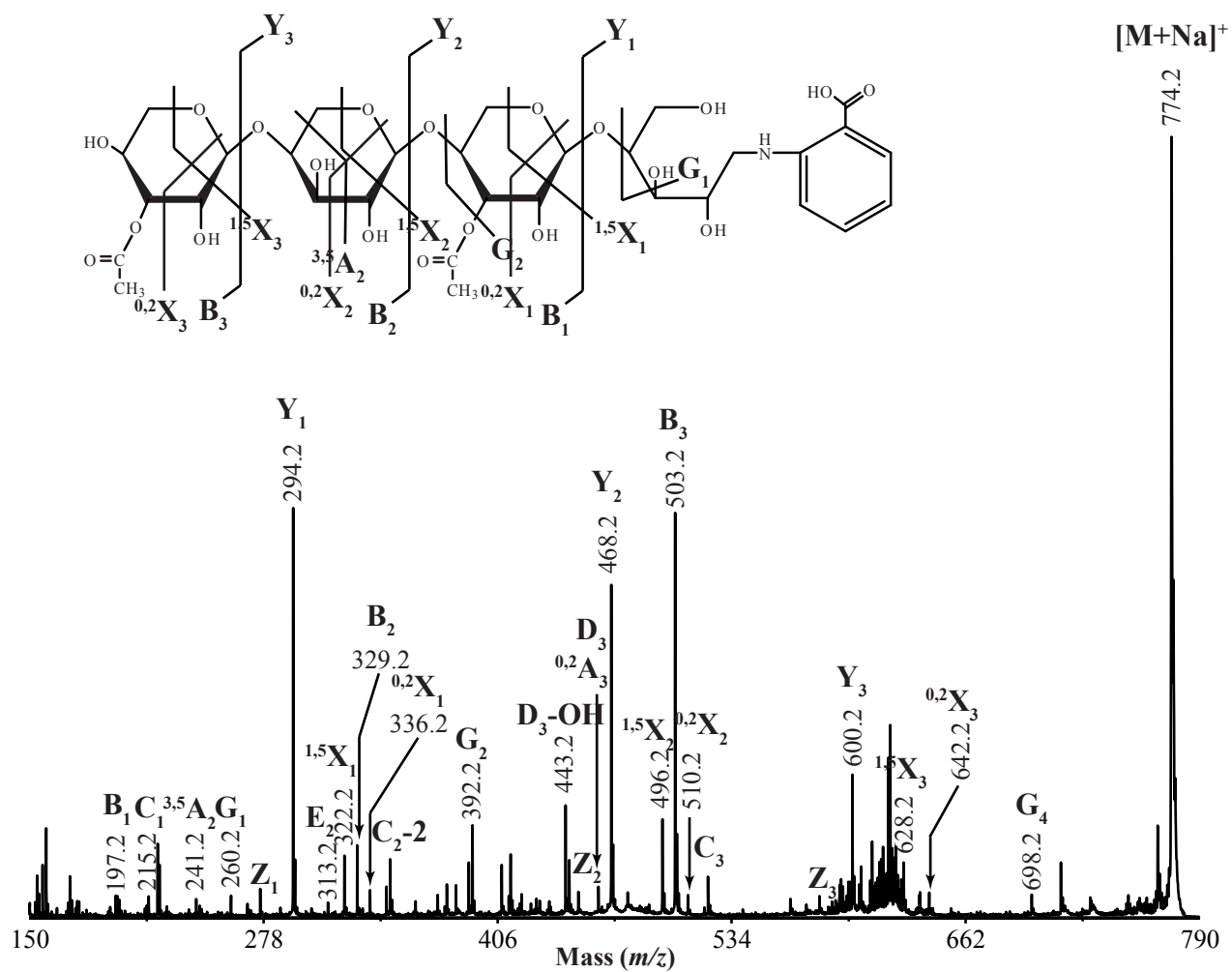

Supplement: Supplementary file 5 — Figure S5. MALDI-CID of Xyl4Ac2 released by CmXyn10B digestion of acetylated xylan from wild-type Arabidopsis stems. [file tpj0079-0492-SD5.pdf]

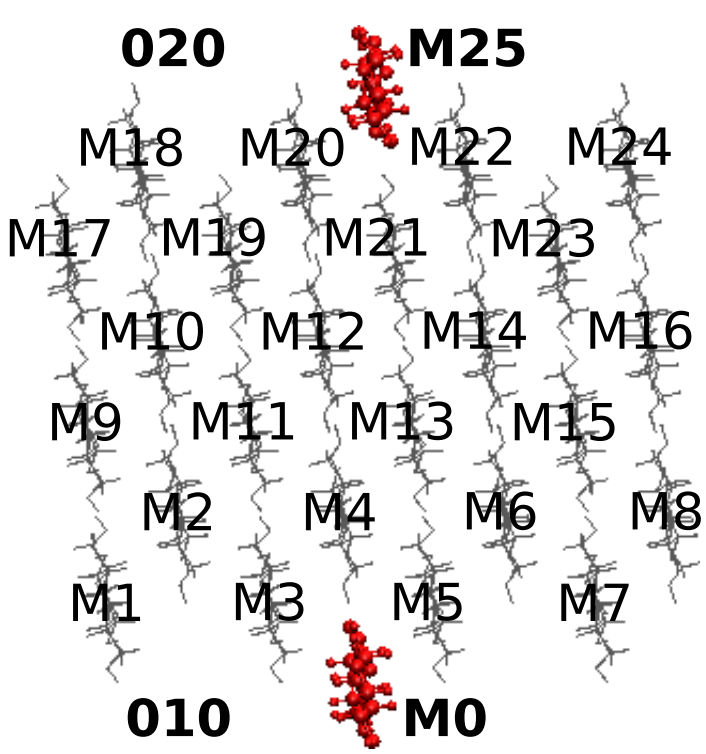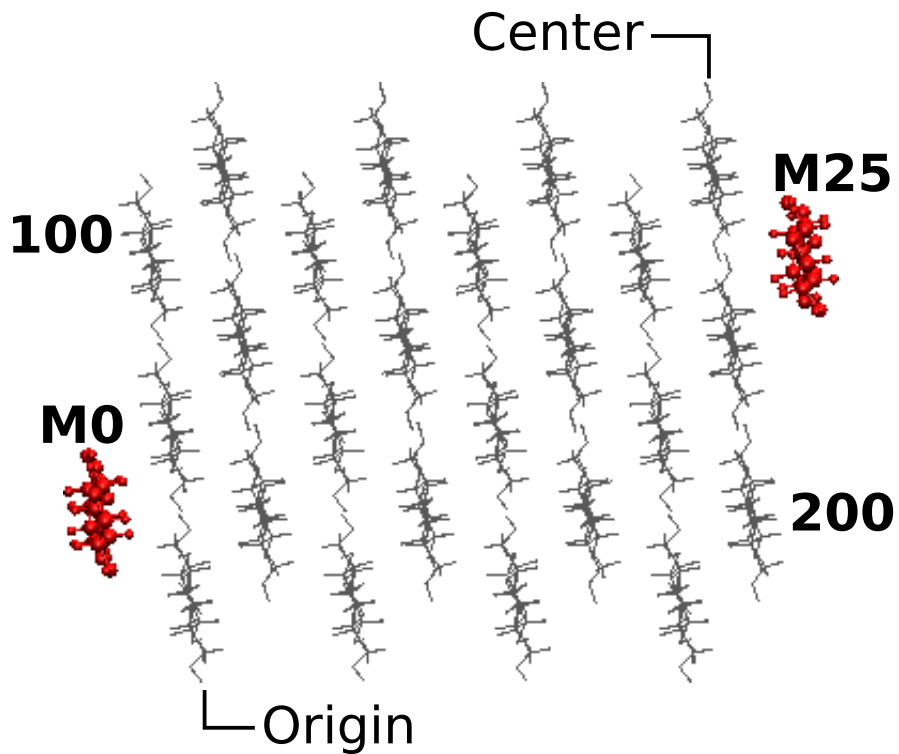

Supplement: Supplementary file 6 — Figure S6. Numbering of individual molecules used in the simulations. [file tpj0079-0492-SD6.pdf]

(a)

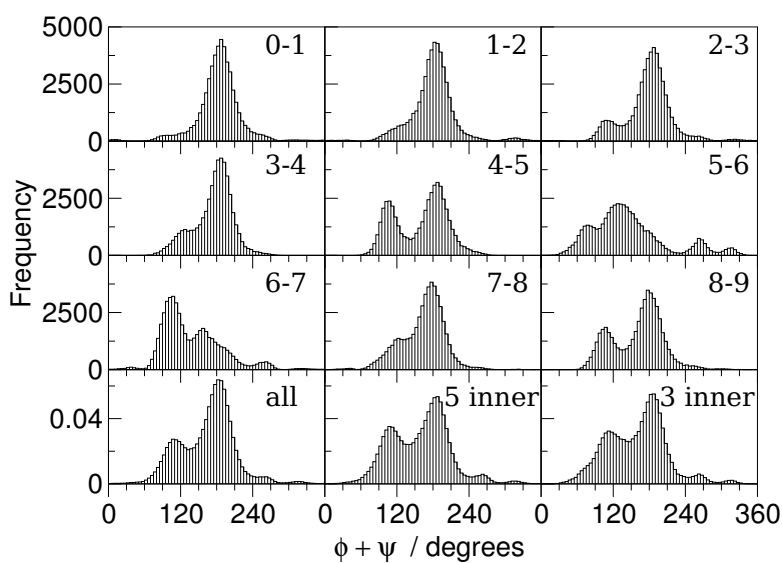

(b)

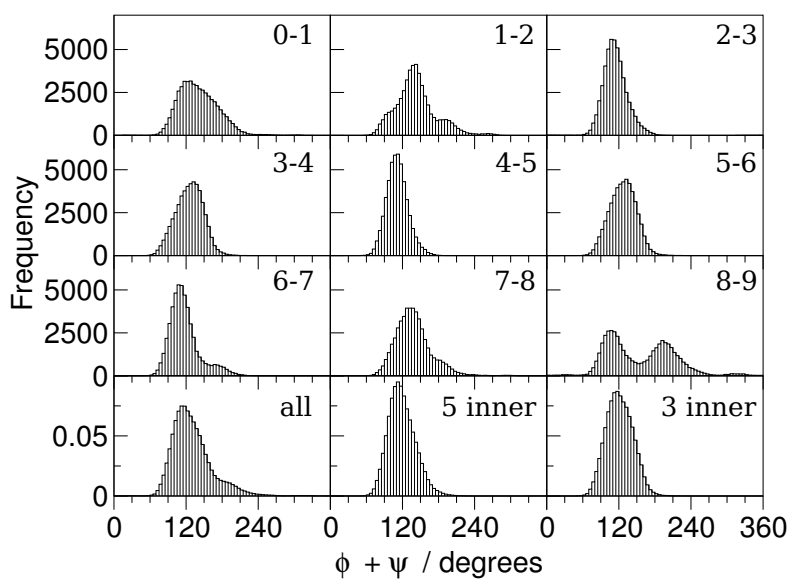

(c)

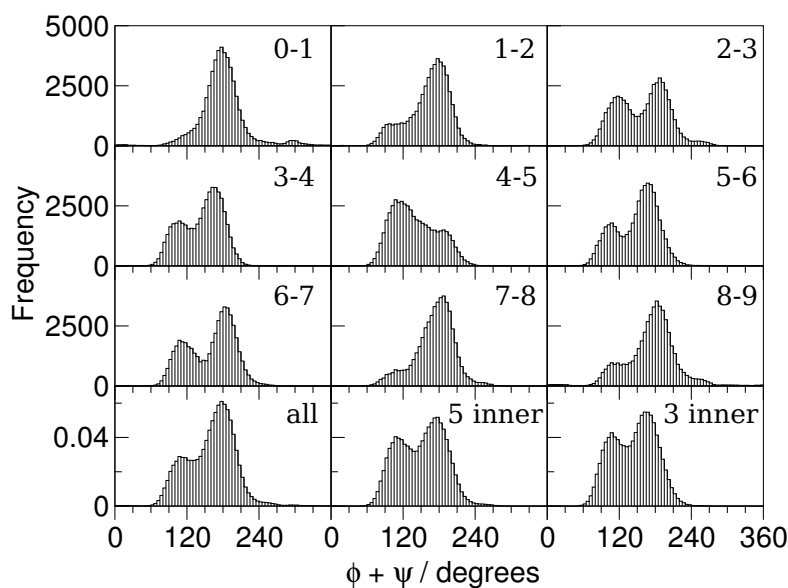

Supplement: Supplementary file 7 — Figure S7. Two MD snapshots showing the slight tilting of the plane of origin chains observed during simulations. [file tpj0079-0492-SD7.pdf]

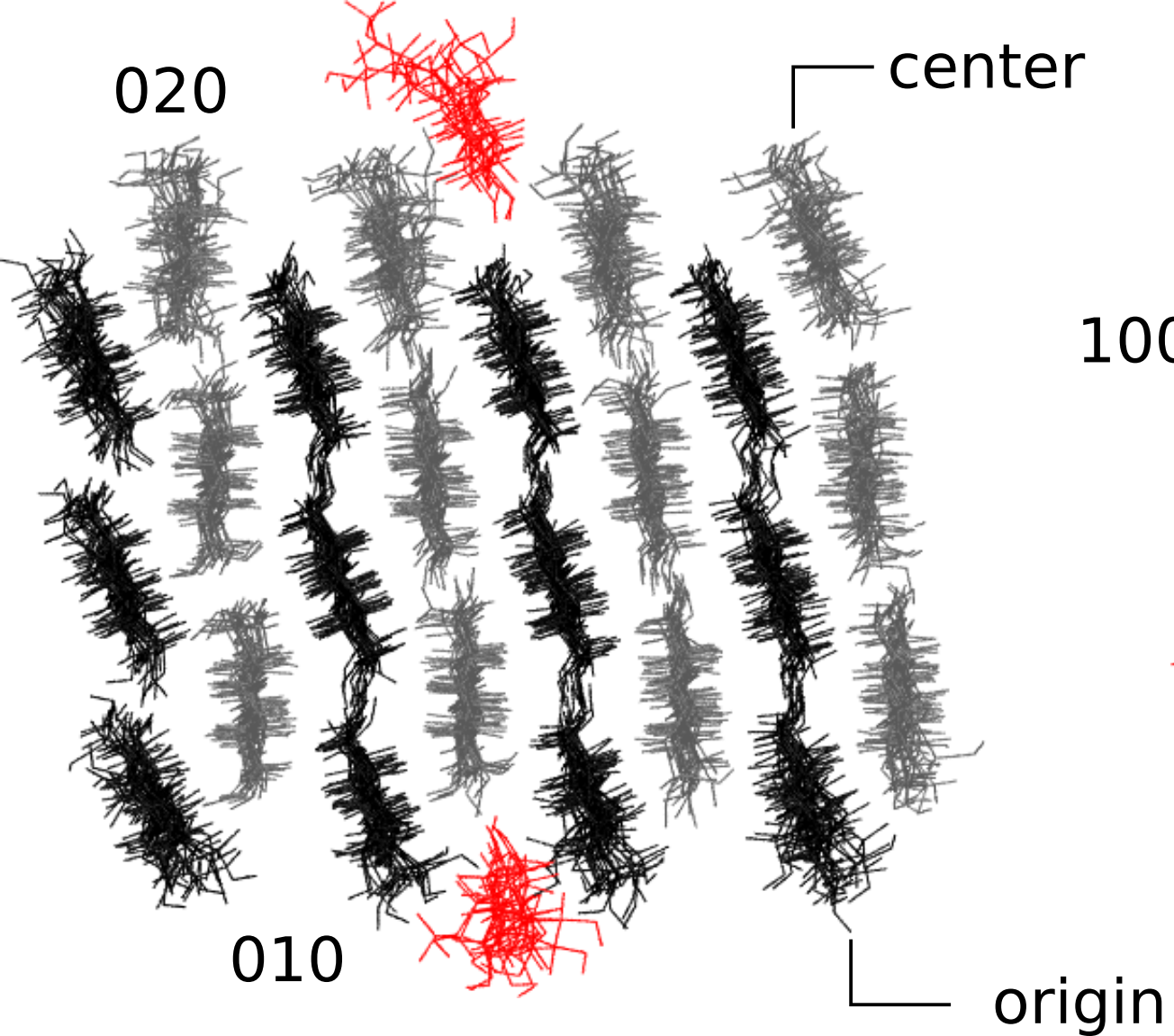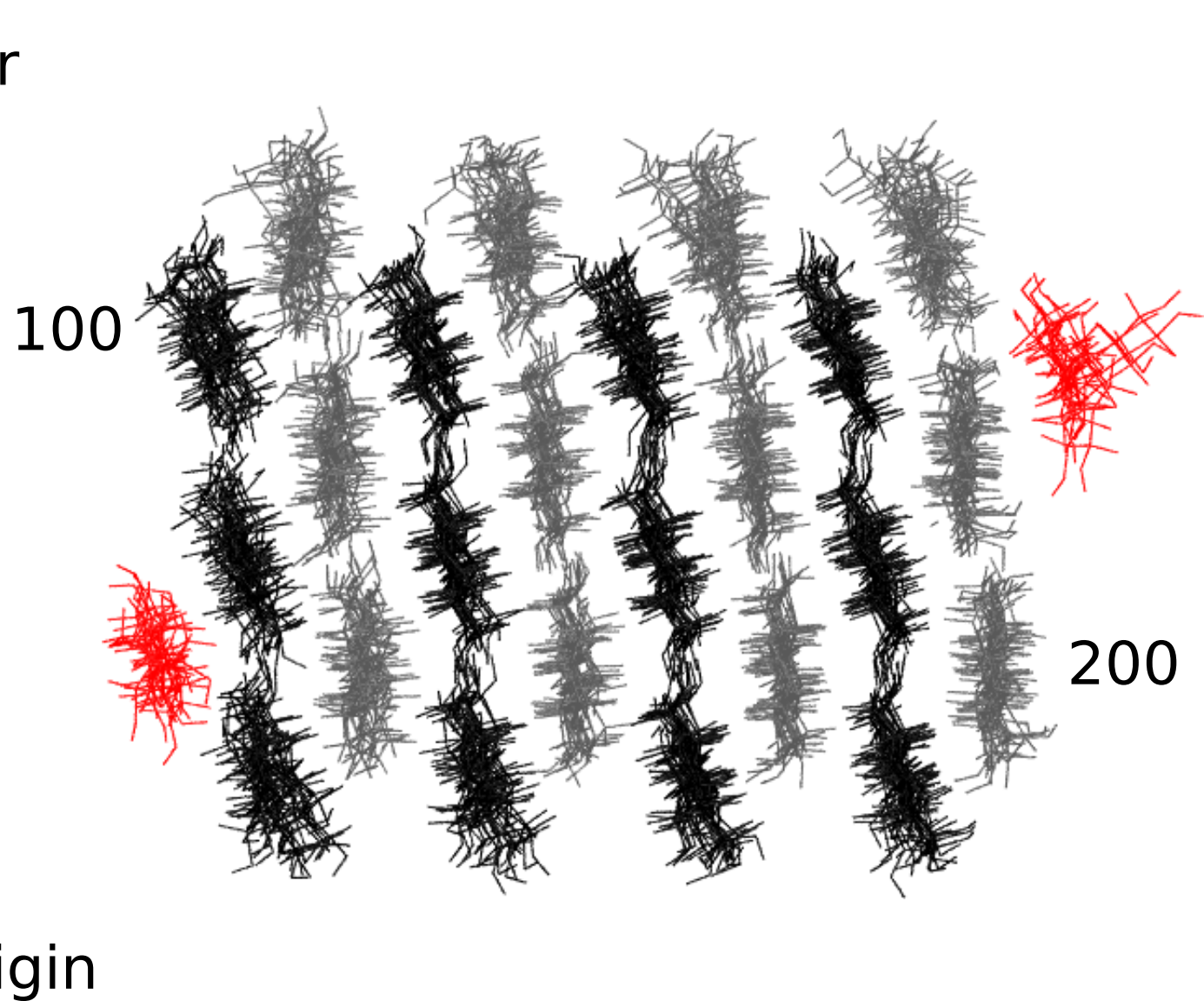

Supplement: Supplementary file 8 — Figure S8. Histograms showing the distribution of glycosidic dihedral angles Φ + Ψ between adjacent xylose residues of unsubstituted xylan DP10. [file tpj0079-0492-SD8.pdf]
