# Supplementary material for: The pattern of xylan acetylation suggests xylan may interact with cellulose microfibrils as a twofold helical screw in the secondary plant cell wall of Arabidopsis thaliana
Source: Plant J. 2014 Jun 6;79(3):492–506. doi: 10.1111/tpj.12575 (PMC4140553; doi:10.1111/tpj.12575)
Supplement: Supplementary file 9 — Table S1. 1H and 13C NMR assignments of acetylated gux1 gux2 xylan at 25°C in D2O. [file tpj0079-0492-SD9.docx]

| Residue |  | Assignment | | | | | |
| --- | --- | --- | --- | --- | --- | --- | --- |
|  |  | 1 | 2 | 3 | 4 | 5 | Me |
| 2-OAcXyl | ^1^H | 4.683 | 4.682 | 3.785 | 3.863 | 3.419, 4.142 | 2.163 |
|  | ^13^C | 100.62 | 74.20 | 72.23 | 76.89 | 63.58 | 21.24 |
| 3-OAcXyl | ^1^H | 4.559 | 3.467 | 4.983 | 3.937 | 3.464, 4,129 | 2.153 |
|  | ^13^C | 102.22 | 71.70 | 76.06 | 76.32 | 63.81 | 21.40 |
| 2,3-OAcXyl | ^1^H | 4.815 | 4.815 | 5.167 | 4.043 | 3.524,4.194 | 2.106 |
|  | ^13^C | 100.14 | 72.21 | 73.72 | 76.07 | 63.59 | ~21.08 |
| X2-**X**-X2 | ^1^H | 4.474 | 3.282 | 3.541 | 3.780 | 3.363, 4.094 |  |
|  | ^13^C | 102.53 | 73.54 | 74.41 | 77.20 | 63.68 |  |
| X2-**X**-X3 | ^1^H | 4.395 | 3.175 | 3.478 | 3.688 | 3.254, 3.986 |  |
|  | ^13^C | 103.47 | 73.49 | 74.18 | 77.70 | 63.69 |  |
| X3-**X**-X2 | ^1^H | 4.487 | 3.283 | 3.555 | 3.783 | n.a. |  |
|  | ^13^C | 102.65 | 73.48 | 74.43 | 77.19 | n.a. |  |
| X3-**X**-X3 | ^1^H | 4.426 | 3.197 | 3.521 | 3.766 | 3.363, 4.049 |  |
|  | ^13^C | 103.44 | 73.56 | 74.34 | 77.12 | 63.75 |  |
| X?-**X**-X23 | ^1^H | 4.453 |  |  |  | 3.422, 3.990 |  |
|  | ^13^C | 102.51 |  |  |  | 63.70 |  |
