# Supplementary material for: The pattern of xylan acetylation suggests xylan may interact with cellulose microfibrils as a twofold helical screw in the secondary plant cell wall of Arabidopsis thaliana
Source: Plant J. 2014 Jun 6;79(3):492–506. doi: 10.1111/tpj.12575 (PMC4140553; doi:10.1111/tpj.12575)
Supplement: Supplementary file 10 — Table S2. Interaction energies between xylans and cellulose. [file tpj0079-0492-SD10.docx]

| Surface | Molecule | Electrostatic | van der Waals | Total Energy |
| --- | --- | --- | --- | --- |
| 010 | xylan | -70 ± 12 | -77 ± 5 | -150 ± 11 |
|  | acetylxylan | -70 ± 15 | -79 ± 6 | -150 ± 16 |
|  | glucuronoxylan | -60 ± 16 | -79 ± 5 | -140 ± 16 |
| 020 | xylan | -40 ± 16 | -50 ± 18 | -90 ± 31 |
|  | acetylxylan | -60 ± 16 | -70 ± 15 | -120 ± 27 |
|  | glucuronoxylan | -50 ± 17 | -74 ± 9 | -120 ± 20 |
| 100 | xylan | -40 ± 10 | -72 ± 7 | -110 ± 13 |
|  | acetylxylan | -30 ± 14 | -60 ± 17 | -80 ± 30 |
|  | glucuronoxylan | -90 ± 30 | -70 ± 12 | -170 ± 38 |
| 200 | xylan | -50 ± 13 | -53 ± 9 | -100 ± 18 |
|  | acetylxylan | -30 ± 18 | -40 ± 20 | -70 ± 36 |
|  | glucuronoxylan | -60 ± 18 | -59 ± 7 | -120 ± 21 |
