# Supplementary material for: The pattern of xylan acetylation suggests xylan may interact with cellulose microfibrils as a twofold helical screw in the secondary plant cell wall of Arabidopsis thaliana
Source: Plant J. 2014 Jun 6;79(3):492–506. doi: 10.1111/tpj.12575 (PMC4140553; doi:10.1111/tpj.12575)
Supplement: Supplementary file 11 — Table S3. Interaction energies between xylans and water. [file tpj0079-0492-SD11.docx]

| Surface | Molecule | Electrostatic | Van der Waals | Total Energy |
| --- | --- | --- | --- | --- |
| 010 | xylan | -170 ± 18 | -39 ± 6 | -210 ± 16 |
|  | acetylxylan | -200 ± 22 | -57 ± 7 | -250 ± 22 |
|  | glucuronoxylan | -590 ± 50 | -35 ± 8 | -620 ± 48 |
| 020 | xylan | -240 ± 46 | -50 ± 10 | -290 ± 51 |
|  | acetylxylan | -210 ± 38 | -62 ± 9 | -270 ± 42 |
|  | glucuronoxylan | -600 ± 55 | -37 ± 9 | -640 ± 54 |
| 100 | xylan | -200 ± 23 | -41 ± 7 | -240 ± 22 |
|  | acetylxylan | -260 ± 42 | -70 ± 10 | -320 ± 46 |
|  | glucuronoxylan | -550 ± 58 | -38 ± 9 | -590 ± 58 |
| 200 | xylan | -240 ± 27 | -47 ± 8 | -290 ± 27 |
|  | acetylxylan | -280 ± 39 | -70 ± 12 | -360 ± 45 |
|  | glucuronoxylan | -620 ± 49 | -40 ± 10 | -660 ± 48 |
| No crystallite | Xylan in water | -320 ± 24 | -71 ± 8 | -390 ± 21 |
