# Supplementary material for: The pattern of xylan acetylation suggests xylan may interact with cellulose microfibrils as a twofold helical screw in the secondary plant cell wall of Arabidopsis thaliana
Source: Plant J. 2014 Jun 6;79(3):492–506. doi: 10.1111/tpj.12575 (PMC4140553; doi:10.1111/tpj.12575)
Supplement: Supplementary file 12 — Table S4. Glucuronoxylan–cellulose and glucuronoxylan–water interaction energies. [file tpj0079-0492-SD12.docx]

|  | Molecule | Electrostatic | Van der Waals | Total Energy |
| --- | --- | --- | --- | --- |
| Cellulose | **xylan** | **-70 ± 12** | **-77 ± 5** | **-150 ± 11** |
|  | glucuronoxylan | -60 ± 16 | -79 ± 5 | -140 ± 16 |
|  | **main chain** | **-70 ± 14** | **-77 ± 5** | **-150 ± 13** |
|  | GlcA | +3 ± 7 | -2.3 ± 0.6 | +1 ± 7 |
| Water | **xylan** | **-170 ± 18** | **-39 ± 6** | **-210 ± 16** |
|  | glucuronoxylan | -590 ± 50 | -35 ± 8 | -620 ± 48 |
|  | **main chain** | **-180 ± 18** | **-37 ± 6** | **-210 ± 16** |
|  | GlcA | -410 ± 45 | +2 ± 6 | -410 ± 44 |
