# Supplementary material for: The pattern of xylan acetylation suggests xylan may interact with cellulose microfibrils as a twofold helical screw in the secondary plant cell wall of Arabidopsis thaliana
Source: Plant J. 2014 Jun 6;79(3):492–506. doi: 10.1111/tpj.12575 (PMC4140553; doi:10.1111/tpj.12575)
Supplement: Supplementary file 13 — Table S5. Acetylxylan–cellulose and acetylxylan–water interaction energies. [file tpj0079-0492-SD13.docx]

|  | Molecule | Electrostatic | Van der Waals | Total Energy |
| --- | --- | --- | --- | --- |
| Cellulose | **xylan** | **-70 ± 12** | **-77 ± 5** | **-150 ± 11** |
|  | acetylxylan | -70 ± 15 | -79 ± 6 | -150 ± 16 |
|  | **main chain** | **-60 ± 15** | **-75 ± 6** | **-130 ± 16** |
|  | acetyls | -13 ± 6 | -4 ± 1 | -16 ± 6 |
| Water | **xylan** | **-170 ± 18** | **-39 ± 6** | **-210 ± 16** |
|  | acetylxylan | -200 ± 22 | -57 ± 7 | -250 ± 22 |
|  | **main chain** | **-160 ± 20** | **-42 ± 6** | **-200 ± 20** |
|  | acetyls | -40 ± 12 | -15 ± 4 | -50 ± 12 |
