# Supplementary material for: The pattern of xylan acetylation suggests xylan may interact with cellulose microfibrils as a twofold helical screw in the secondary plant cell wall of Arabidopsis thaliana
Source: Plant J. 2014 Jun 6;79(3):492–506. doi: 10.1111/tpj.12575 (PMC4140553; doi:10.1111/tpj.12575)
Supplement: Supplementary file 14 — Table S6. Xylan–cellulose hydrogen bonding statistics for xylans adsorbed on the (010) face of cellulose. [file tpj0079-0492-SD14.docx]

| Xylosyl | Donor | Acceptor | Xylan | Acetylxylan | Glucuronoxylan |
| --- | --- | --- | --- | --- | --- |
| 0 | Xyl0 O2 | Glc5 O6 | 30.9 | 25.2 | 42.2 |
|  | Glc5 O6 | Xyl0 O2 | 49.9 | 13.8 | 18.3 |
|  | Xyl0 O3 | Glc5 O6 | 16.8 | 9.7 | 30.7 |
|  | Glc5 O6 | Xyl0 O3 | 2.6 | 7.0 | 7.0 |
| 2 | Xyl2 O2 | Glc7 O6 | 91.8 | 86.1 | 86.1 |
|  | Glc7 O6 | Xyl2 O2 | 0.3 | 2.8 | 1.3 |
|  | Xyl2 O3 | Glc7 O6 | 6.8 | 4.7 | 7.4 |
|  | Glc7 O6 | Xyl2 O3 | 9.8 | 7.5 | 16.8 |
| 4 | Xyl4 O2 | Glc9 O6 | 91.6 | 94.2 | 85.0 |
|  | Glc9 O6 | Xyl4 O2 | 3.2 | 0.5 | 1.4 |
|  | Xyl4 O3 | Glc9 O6 | 2.7 | 3.7 | 2.3 |
|  | Glc9 O6 | Xyl4 O3 | 11.5 | 12.9 | 20.8 |
| 6 | Xyl6 O2 | Glc11 O6 | 87.6 | 60.7 | 88.6 |
|  | Glc11 O6 | Xyl6 O2 | 0.7 | 5.4 | 0.9 |
|  | Xyl6 O3 | Glc11 O6 | 4.4 | 1.6 | 4.1 |
|  | Glc11 O6 | Xyl6 O3 | 13.5 | 38.7 | 15.1 |
| 8 | Xyl8 O2 | Glc13 O6 | 87.9 | 57.9 | 81.3 |
|  | Glc13 O6 | Xyl8 O2 | 2.3 | 2.2 | 3.6 |
|  | Xyl8 O3 | Glc13 O6 | 1.9 | 4.8 | 1.7 |
|  | Glc13 O6 | Xyl8 O3 | 13.3 | 20.9 | 27.7 |
