# Supplementary material for: The pattern of xylan acetylation suggests xylan may interact with cellulose microfibrils as a twofold helical screw in the secondary plant cell wall of Arabidopsis thaliana
Source: Plant J. 2014 Jun 6;79(3):492–506. doi: 10.1111/tpj.12575 (PMC4140553; doi:10.1111/tpj.12575)
Supplement: Supplementary file 15 — Table S7. Xylan–cellulose hydrogen bonding statistics for xylans adsorbed on the (020) face of cellulose. [file tpj0079-0492-SD15.docx]

| Xylosyl | Donor | Acceptor | Xylan | Acetylxylan | Glucuronoxylan |
| --- | --- | --- | --- | --- | --- |
| 1 | Xyl1 O2 | Glc6 O6 | 8.0 | 42.1 | 53.1 |
|  | Glc6 O6 | Xyl1 O2 | 0.2 | 4.1 | 1.9 |
|  | Xyl1 O3 | Glc6 O6 | 2.3 | 6.1 | 4.8 |
|  | Glc6 O6 | Xyl1 O3 | 0.7 | 21.5 | 23.2 |
| 3 | Xyl3 O2 | Glc8 O6 | 19.7 | 61.9 | 82.5 |
|  | Glc8 O6 | Xyl3 O2 | 2.0 | 1.2 | 1.3 |
|  | Xyl3 O3 | Glc8 O6 | 6.8 | 2.8 | 2.6 |
|  | Glc8 O6 | Xyl3 O3 | 1.5 | 14.1 | 17.7 |
| 5 | Xyl5 O2 | Glc10 O6 | 64.1 | 94.2 | 88.4 |
|  | Glc10 O6 | Xyl5 O2 | 0.7 | 0.3 | 3.3 |
|  | Xyl5 O3 | Glc10 O6 | 6.1 | 6.0 | 2.3 |
|  | Glc10 O6 | Xyl5 O3 | 4.5 | 16.3 | 34.5 |
| 7 | Xyl7 O2 | Glc12 O6 | 56.8 | 86.5 | 91.5 |
|  | Glc12 O6 | Xyl7 O2 | 0.6 | 1.1 | 1.3 |
|  | Xyl7 O3 | Glc12 O6 | 12.4 | 4.5 | 3.3 |
|  | Glc12 O6 | Xyl7 O3 | 24.6 | 32.3 | 21.5 |
| 9 | Xyl9 O2 | Glc14 O6 | 19.5 | 46.4 | 86.5 |
|  | Glc14 O6 | Xyl9 O2 | 1.7 | 1.8 | 1.8 |
|  | Xyl9 O3 | Glc14 O6 | 2.0 | 2.5 | 8.3 |
|  | Glc14 O6 | Xyl9 O3 | 3.0 | 22.1 | 27.1 |
