# Supplementary material for: The pattern of xylan acetylation suggests xylan may interact with cellulose microfibrils as a twofold helical screw in the secondary plant cell wall of Arabidopsis thaliana
Source: Plant J. 2014 Jun 6;79(3):492–506. doi: 10.1111/tpj.12575 (PMC4140553; doi:10.1111/tpj.12575)
Supplement: Supplementary file 16 — Table S7. Xylan–cellulose hydrogen bonding statistics for xylans adsorbed on the (020) face of cellulose. [file tpj0079-0492-SD16.docx]

Supporting information figure legends

Figure S1. Model of acetylated xylan interacting with 36 chain cellulose microfibril with hexagonal cross section. Xylan DP10, acetylated on alternate xylosyl residues, is placed on hydrophilic surfaces 1-10 and 110.

Figure S2. H3/C3 regions of a 2D ^13^C HSQC spectrum. A gradient-selective ^13^C HSQC experiment incorporating a long recovery period was recorded for the purpose of quantifying the degree of the different acetylations.

Figure S3. MALDI-CID of Xyl_4_Ac_3_ released by *Cm*Xyn10B from acetylated *gux1gux2* stem xylan, labeled with 2-AA and separated by HILIC showed the presence of xylose di-substituted at O2 and O3 with acetate

Figure S4. MALDI-CID on the Xyl_4_Ac_2_ and Xyl_6_Ac_3_ released by *Ec*Xyn30 from acetylated *gux1gux2* stem xylan. (a) MALDI-CID of the Xyl_4_Ac_2_, (b) MALDI-CID of Xyl_6_Ac_3_.

Figure S5. MALDI-CID of Xyl_4_Ac_2_ released by *Cm*Xyn10B digestion of acetylated xylan from wild-type Arabidopsis stems.

Figure S6. Numbering of individual molecules in 24 glucan chain crystallites used in the simulations and the two xylan chains M0 and M25 (red). The figure shows 010 and 020 hydrophilic and 100 and 200 hydrophobic surfaces, and examples of the center and origin planes.

Figure S7. Two MD snapshots showing the slight tilting of the plane of origin chains observed during simulations.

Figure S8. Continuation of experiment shown on Figure 8. Xylan in 3_1_ and 2_1_ fold screw conformations. Histograms showing the distribution of glycosidic dihedral angles Φ + Ψ between adjacent xylose residues of unsubstituted xylan of DP10. Numbers refer to xylose residues.

(a) xylan on cellulose face 020.

(b) xylan on cellulose face 100.

(c) xylan on cellulose face 200.
