# Supplementary material for: The pattern of xylan acetylation suggests xylan may interact with cellulose microfibrils as a twofold helical screw in the secondary plant cell wall of Arabidopsis thaliana
Source: Plant J. 2014 Jun 6;79(3):492–506. doi: 10.1111/tpj.12575 (PMC4140553; doi:10.1111/tpj.12575)
Supplement: Supplementary file 17 — Table S7. Xylan–cellulose hydrogen bonding statistics for xylans adsorbed on the (020) face of cellulose. [file tpj0079-0492-SD17.docx]

Supporting information table legends

Table S1. ^1^H and ^13^C NMR assignments of acetylated *gux1gux2* xylan at 25 °C in D_2_O.

Table S2. Interaction energies between xylans and cellulose for each simulated system in kcal/mol.

Table S3. Interaction energies between xylans and water for each simulated system in kcal/mol.

Table S4. Glucuronoxylan-cellulose and glucuronoxylan-water interaction energies in kcal/mol from MD simulations, separated in terms of the xylosyl main chain and decorating residue contributions. Interaction energies for unsubstituted xylan with cellulose and water are shown for comparison. Adsorption face is 010.

Table S5. Acetylxylan-cellulose and acetylxylan-water interaction energies in kcal/mol from MD simulations, separated in terms of the xylosyl main chain and decorating residue contributions. Interaction energies for substituted xylan with cellulose and water are shown for comparison. Adsorption face is 010.

Table S6. Xylan-cellulose hydrogen bonding statistics for xylans adsorbed on the 010 face of cellulose, given as percentage of simulation time a hydrogen bond between the indicated atomic pair is identified.

Table S7. Xylan-cellulose hydrogen bonding statistics for xylans adsorbed on the 020 face of cellulose, given as percentage of simulation time a hydrogen bond between the indicated atomic pair is identified.
